# Linearly Implicit Finite Element Methods Approximating the Solution to the Nonlinear Schrödinger Equation with a Schamel-Type Nonlinearity

**DOI:** 10.1007/s10915-026-03328-2

**Published:** 2026-05-26

**Authors:** Panagiotis Paraschis, Georgios E. Zouraris

**Affiliations:** 1 Faculty of Mathematics, University of Vienna, Oscar-Morgestern-Platz 1, A-1090 Vienna, Austria; 2 Institute of Analysis and Scientific Computing, Vienna University of Technology, Wiedner Hauptstraße 8-10, A-1040 Vienna, Austria; 3 Department of Mathematics and Applied Mathematics, Division of Applied Mathematics: Differential Equations and Numerical Analysis, University of Crete, GR-700 13 Voutes Campus, Heraklion, Crete Greece

**Keywords:** Nonlinear Schrödinger equation, Schamel nonlinearity, Linearly implicit time-stepping, Finite element method, Stability, Error estimates, Convergence, 65M15, 65M60, 81Q05

## Abstract

We consider an initial- and Dirichlet boundary- value problem for a nonlinear Schrödinger equation of the form $u_t=\,\textrm{i}\,{\varDelta }u+\textrm{i}\,V\,u+\textrm{i}\,\mu \,|u|^{\beta }\,u+f$ over $[0,T]\times {\varOmega }$, where $T>0$, ${\varOmega }\subset {\mathbb {R}}^d$ for $d\in \{1,2,3\}$, $\beta \in (0,1)$, *V* is a real-valued time-independent potential and $\mu$ is a nonzero real number. The solution to the problem is approximated by the Linearized Backward Euler finite element (LBEFE) method which is dissipative and the Linearized Crank–Nicolson finite element (LCNFE) one which is conservative. Letting $\tau$ be the time-step and *h* be the width of the finite element partition of the space domain, we provide an optimal order $O(\tau +h^2)$ error estimate in the $L^2$ norm for both methods, and an $O(\tau ^{\alpha }+h)$ error estimate in the $H^1$ norm, where $\alpha =\frac{3}{4}$ in the (LBEFE) method and $\alpha =\frac{1}{2}$ in the (LCNFE) one. For $d=1$, no CFL conditions are imposed, while for $d=2$ or 3, a mesh condition of the form $\big (h^{\frac{2-d}{2}}\,|\ln (h)|^{\frac{d-1}{d}} \,\tau ^{\alpha }+h^{2-\frac{d}{2}}\big )=O(1)$ is required. Finally, with results from numerical experiments, we investigate the performance of the methods proposed and analyzed.

## Introduction

### Formulation of the Problem

Let $T>0$ be a final time, $d\in \{1,2,3\}$, ${\varOmega }\subset {\mathbb {R}}^d$ be a bounded convex domain with smooth or polygonal boundary $\partial {\varOmega }$, $\beta \in (0,1)$, $\mu$ be a nonzero real number and ${\mathfrak g}:{\mathbb {R}}^2\rightarrow {\mathbb {C}}$ be a complex-valued function defined by

1.1 $$\begin{aligned} {\mathfrak g}(x)=\textrm{i}\,\mu \,|x|_{\scriptscriptstyle \mathsf E}^{\beta }\,(x_1+\textrm{i}\,x_2) \quad \forall \,x\in {\mathbb {R}}^2, \end{aligned}$$

where $|\cdot |_{\scriptscriptstyle \mathsf E}$ denotes the usual Euclidean norm on ${\mathbb {R}}^2$. Then, we consider the model initial- and Dirichlet boundary- value problem of finding a function $u:[0,T]\times {\overline{{\varOmega }}}\rightarrow {\mathbb {C}}$ such that

1.2 $$\begin{aligned} u_t&=\,\textrm{i}\,{\varDelta }u+\textrm{i}\,V\,u+g(u)+f\quad \forall \,(t,x)\in (0,T]\times {\varOmega }, \end{aligned}$$

1.3 $$\begin{aligned}&u(t,\cdot )|_{\scriptscriptstyle \partial D}=\,0\qquad \forall \,t\in (0, T], \end{aligned}$$

1.4 $$\begin{aligned} u(0,x)&=\,u_0(x)\qquad \forall \,x\in {\overline{{\varOmega }}}, \end{aligned}$$

where $u_0:{\overline{{\varOmega }}}\rightarrow {\mathbb {C}}$ is a smooth complex-valued function with $u_0|_{\scriptscriptstyle \partial {\varOmega }}=0$, $f:[0,T]\times {\overline{{\varOmega }}}\rightarrow {\mathbb {C}}$, $V:{\overline{{\varOmega }}}\rightarrow {\mathbb {R}}$ a bounded, real-valued function, and $g:{\mathbb {C}}\rightarrow {\mathbb {C}}$ is a complex-valued function given by

1.5 $$\begin{aligned} g(z):={\mathfrak g}(\textsf{Re}(z),\textsf{Im}(z))=\textrm{i}\,\mu \,|z|^{\beta }\,z \quad \forall \,z\in {\mathbb {C}}. \end{aligned}$$

For $\beta =\frac{1}{2}$, we obtain the *Schamel-nonlinear Schrödinger equation* which appears as a mathematical model for the propagation of waves in a multicomponent dense plasma (see, e.g., [18, 30, 31]), while the Schamel-nonlinearity has been proposed earlier, in the context of the Korteweg de Vries equation, for the investigation of one-dimensional ion-acoustic waves due to resonant electrons [34]. Moreover, a Schamel-type nonlinearity, with $\beta =\tfrac{2}{3}$, appears in [7] in a Schrödinger-Poisson-X$\alpha$ model.

We refer the reader to [13, 28] and [40], and the references therein, for mathematical results on the problem above. To the knowledge of the authors, the existing bibliography on the numerical approximation of the problem above is limited and includes time-splitting methods (see [15, 23]), time-splitting pseudospectral methods (see, e.g. [5, 6, 8, 24]), the Backward Euler finite difference method [22], a linearized Crank–Nicolson finite difference method [30], and space-time finite element methods [2]. Here, we are interested in the approximation of the solution to the problem by linearly implicit time-stepping methods, because they avoid the use of solvers of nonlinear systems of algebraic equations, and by the usual conforming finite element method in space, because, in contrast to the finite difference method, it is applicable to general domains, and, in contrast to the splitting methods, is independent of the domain-dependent spectrum of the Laplace operator.

### Notation and Preliminaries

Hereafter, we denote by $L^{\infty }({\varOmega })$ the space consisting of all Lebesgue measurable complex-valued functions which have their essential supremum bounded on ${\varOmega }$, provided with the standard norm $|v|_{\infty }:= \text {ess\,sup}_{\scriptscriptstyle {\varOmega }}|v|$ for $v\in L^{\infty }({\varOmega })$. Also, we denote by $L^2({\varOmega })$ the space consisting of all Lebesgue measurable complex-valued functions which have the absolute value of its second power integrable on ${\varOmega }$ with respect to Lebesgue’s measure *dx*, provided with the standard norm $\Vert v\Vert := \left( \int _{\scriptscriptstyle {\varOmega }}|v(x)|^2\,dx\right) ^{\scriptscriptstyle 1/2}$ for $v\in L^2({\varOmega })$, which is derived by the usual inner product $(v_1,v_2):=\int _{\scriptscriptstyle {\varOmega }}v_1(x)\,{\overline{v_2}(x)}\,dx$ for $v_1,v_2\in L^2({\varOmega })$. Let ${\mathbb {N}}_0$ be the set of all non-negative integers and $\kappa \in {\mathbb {N}}_0$. We denote by $H^{\kappa }({\varOmega })$ the Sobolev space of complex-valued functions which belong, along with their generalized derivatives up to order $\kappa$, to $L^2({\varOmega })$, and by $\Vert \cdot \Vert _{\kappa }$ its usual norm, i.e. $\Vert v\Vert _{\kappa }:=\left( \, \sum _{\beta _{\star }\in {\mathbb {N}}^d_0,\,|\beta _{\star }|\le \kappa } \Vert \partial ^{\beta _{\star }}v\Vert ^2\,\right) ^{\scriptscriptstyle 1/2}$ for $v\in H^{\kappa }({\varOmega })$. Also, by $H_0^1({\varOmega })$ we denote the subspace of $H^1({\varOmega })$ consisting of functions which vanish at the boundary $\partial {\varOmega }$ of ${\varOmega }$ in the sense of trace and we simplify the notation by setting ${\mathbb {H}}^s({\varOmega }):=H^s({\varOmega })\cap H_0^1({\varOmega })$ for $s\ge 1$.

For later use, we recall the following useful identity

1.6 $$\begin{aligned} \textsf{Re}\left[ (v_2-v_1,v_2)\right] =\tfrac{1}{2}\,\left( \,\Vert v_2\Vert ^2-\Vert v_1\Vert ^2 +\Vert v_2-v_1\Vert ^2\,\right) \quad \forall \,v_1,v_2\in L^2({\varOmega }), \end{aligned}$$

and we show below that *g* enjoys a very useful global Lipschitz-type inequality.

#### Lemma 1.1

Let ${\mathfrak g}$ and *g* be given by (1.1) and (1.5), respectively. Then, ${\mathfrak g}\in C^1({\mathbb {R}}^2)$ and it holds that

1.7 $$\begin{aligned} |g(v)-g(w)|\le \,2\,|\mu |\,(1+\beta ) \,(|v|^{\beta }+|w|^{\beta })\,|v-w| \quad \forall \,v,w\in {\mathbb {C}}. \end{aligned}$$

**Proof**

It is obvious that ${\mathfrak g}(x)=\textrm{i}\,\mu \,\left[ \Phi (x_1,x_2)+\textrm{i}\, \Phi (x_2,x_1)\right]$ for $x\in {\mathbb {R}}^2$, where $\Phi :{\mathbb {R}}^2\rightarrow {\mathbb {R}}$ is a function defined by $\Phi (y)=y_1\,|y|_{\scriptscriptstyle \mathsf E}^\beta$ for $y\in {\mathbb {R}}^2$.

Let $x^{\star }\in {\mathbb {R}}^2$ with $x^{\star }\not =(0,0)$. It is obvious that the first order partial derivatives $\partial _{x_1}\Phi (x^{\star })$ and $\partial _{x_2}\Phi (x^{\star })$ exist and are given by $\partial _{x_1}\Phi (x^{\star })=|x^{\star }|_{\scriptscriptstyle \mathsf E}^{\beta } +\beta \,(x^{\star }_1)^2\,|x^{\star }|_{\scriptscriptstyle \mathsf E}^{\beta -2}$ and $\partial _{x_2}\Phi (x^{\star })=\beta \,x^{\star }_1 \,x^{\star }_2\,|x^{\star }|_{\scriptscriptstyle \mathsf E}^{\beta -2}$. From the latter formulas, we conclude that $\partial _{x_1}\Phi$ and $\partial _{x_2}\Phi$ are continuous on ${\mathbb {R}}^2\backslash \{(0,0)\}$. For $x^{\star }=(0,0)$, we have $\partial _{x_1}\Phi (x^{\star })=\partial _{x_1}\Phi (0,0) =\lim \limits _{\varepsilon \rightarrow 0}\tfrac{\Phi (\varepsilon ,0)-\Phi (0,0)}{\varepsilon } =\lim \limits _{\varepsilon \rightarrow 0}|\varepsilon |^{\beta }=0$ and $\partial _{x_2}\Phi (x^{\star })=\partial _{x_2}\Phi (0,0) =\lim \limits _{\varepsilon \rightarrow 0}\tfrac{\Phi (0,\varepsilon )-\Phi (0,0)}{\varepsilon } =\lim \limits _{\varepsilon \rightarrow 0}0=0$. Observing that $0\le y_1^2\,|y|_{\scriptscriptstyle \mathsf E}^{\beta -2}\le \,|y|_{\scriptscriptstyle \mathsf E}^{\beta }$ and $0\le |y_1|\,|y_2|\,\,|y|_{\scriptscriptstyle \mathsf E}^{\beta -2}\le \,|y|_{\scriptscriptstyle \mathsf E}^{\beta }$ for $y\in {\mathbb {R}}^2$ with $y\not =(0,0)$, we, easily, conclude that $\lim \limits _{y\rightarrow (0,0)}\partial _{x_1}\Phi (y)=0=\partial _{x_1}\Phi (0,0)$ and $\lim \limits _{y\rightarrow (0,0)}\partial _{x_2}\Phi (y)=0=\partial _{x_2}\Phi (0,0)$. Thus, $\Phi \in C^1({\mathbb {R}}^2)$.

Since $|\partial _{x_1}\Phi (y)|\le \,(1+\beta )\,|y|_{\scriptscriptstyle \mathsf E}^{\beta }$ and $|\partial _{x_2}\Phi (y)|\le \,\beta \,|y|_{\scriptscriptstyle \mathsf E}^{\beta }$ for $y\in {\mathbb {R}}^2$, applying the mean value theorem for scalar functions, we get

$$\begin{aligned} \begin{aligned} |\Phi (x)-\Phi (y)|\le&\,\sup _{s\in (0,1)} |\nabla \Phi (s\,x+(1-s)\,y)|_{\scriptscriptstyle \mathsf E}\,|x-y|_{\scriptscriptstyle \mathsf E}\\ \le&\,\sup _{s\in (0,1)}\left( |\partial _{x_1}\Phi (s\,x+(1-s)y)|^2 +|\partial _{x_2}\Phi (s\,x+(1-s)y)|^2\right) ^{\frac{1}{2}} \,|x-y|_{\scriptscriptstyle \mathsf E}\\ \le&\,\sqrt{2}\,(1+\beta ) \,\left( \,|x|_{\scriptscriptstyle \mathsf E} +|y|_{\scriptscriptstyle \mathsf E}\,\right) ^{\beta }\,|x-y|_{\scriptscriptstyle \mathsf E}\\ \le&\,\sqrt{2}\,(1+\beta ) \,\left( \,|x|_{\scriptscriptstyle \mathsf E}^{\beta } +|y|_{\scriptscriptstyle \mathsf E}^{\beta }\,\right) \,|x-y|_{\scriptscriptstyle \mathsf E} \quad \forall \,x,y\in {\mathbb {R}}^2.\\ \end{aligned} \end{aligned}$$

Thus, we have

$$\begin{aligned} \begin{aligned} |{\mathfrak g}(x)-{\mathfrak g}(y)|=&\,|\mu |\, \left[ \left( \Phi (x_1,x_2)-\Phi (y_1,y_2)\right) ^2 +\left( \Phi (x_2,x_1)-\Phi (y_2,y_1)\right) ^2 \right] ^{\frac{1}{2}}\\ \le&\,2\,|\mu |\,(1+\beta ) \,(|x|_{\scriptscriptstyle \mathsf E}^{\beta }+|y|_{\scriptscriptstyle \mathsf E}^{\beta })\,|x-y|_{\scriptscriptstyle \mathsf E} \quad \forall \,x,y\in {\mathbb {R}}^2,\\ \end{aligned} \end{aligned}$$

which, obviously, yields (1.7).

#### Remark 1.1

Observing that $\tfrac{|\partial _{x_1}\Phi (\varepsilon ,0) -\partial _{x_1}\Phi (0,0)|}{|\varepsilon |} =(1+\beta )\,|\varepsilon |^{\beta -1}$ is not bounded for $\varepsilon$ close to zero, we conclude that $\partial _{x_1}\Phi$ could not be a Lipschitz function around (0, 0) and that the second order partial derivative $\partial _{x_1}^2\Phi$ is not defined at (0, 0). Thus, we can not provide function ${\mathfrak g}$ with higher regularity properties over a domain that contains the origin.

Let ${\textsf{S}_h}\subset {\mathbb {H}}^1({\varOmega })\cap C({\overline{{\varOmega }}})$ be a finite element space consisting of functions which are continuous on ${\overline{{\varOmega }}}$ and piecewise linear polynomials over a shape regular partition of ${\varOmega }$ in triangles with maximum diameter *h*. Then, we introduce a discrete Laplacian operator $\varDelta _h:H^1({\varOmega })\rightarrow {\textsf{S}_h}$ by

1.8 $$\begin{aligned} (\varDelta _hw,\chi )=(\nabla {w},\nabla {\chi }) \quad \forall \chi \in {\textsf{S}_h},\quad \forall \,w\in H^1({\varOmega }), \end{aligned}$$

the $L^2({\varOmega })$-projection operator $\textsf{P}_h:L^2({\varOmega })\rightarrow {\textsf{S}_h}$ by

$$\begin{aligned} (\textsf{P}_hw,\chi )=(w,\chi )\quad \forall \,\chi \in {\textsf{S}_h}, \quad \forall \,w\in L^2({\varOmega }), \end{aligned}$$

and the elliptic projection operator $\textsf{R}_h:H^1({\varOmega })\rightarrow {\textsf{S}_h}$ by

$$\begin{aligned} (\nabla \textsf{R}_hw,\nabla \chi )=(\nabla w,\nabla \chi ) \quad \forall \,\chi \in {\textsf{S}_h}, \quad \forall \,w\in H^1({\varOmega }). \end{aligned}$$

It is well-known (see, e.g., Theorem 4.4.20 and Corollary 4.4.24 in [10]) that for the usual Lagrange interpolant ${\mathcal {I}}_h:C({\overline{{\varOmega }}})\rightarrow {\textsf{S}_h}$ there exist positive constants $\textsf{C}_{{\texttt {IP1}}}$ and $\textsf{C}_{{\texttt {IP2}}}$, independent of the partition of ${\varOmega }$, such that

1.9 $$\begin{aligned} |{\mathcal {I}}_hw-w|_{\infty }\le \,\textsf{C}_{\texttt {IP1}} \,h^{2-\frac{d}{2}}\,\Vert w\Vert _2 \quad \,\forall \,w\in {\mathbb {H}}^2({\varOmega }) \end{aligned}$$

and

1.10 $$\begin{aligned} \Vert {\mathcal {I}}_hw-w\Vert +h\,\Vert {\mathcal {I}}_hw-w\Vert _1 \le \,\textsf{C}_{\texttt {IP2}}\,h^2\,\Vert w\Vert _2 \quad \,\forall \,w\in {\mathbb {H}}^2({\varOmega }). \end{aligned}$$

In light of (1.10), it is well-known (see, e.g. Theorem 1.1 in [38]) that there exists a constant $\textsf{C}_{{\texttt {EP1}}}>0$, independent of the partition of ${\varOmega }$, such that

1.11 $$\begin{aligned} \Vert \textsf{R}_h{w}-w\Vert +h\,\Vert \textsf{R}_h{w}-w\Vert _1 \le \,\textsf{C}_{\texttt {EP1}}\,h^2\,\Vert w\Vert _2 \quad \,\forall \,w\in {\mathbb {H}}^2({\varOmega }). \end{aligned}$$

Also, we assume that the triangulation of ${\varOmega }$ is quasi-uniform and thus (see, e.g., Theorem 4.5.11 in [10], Remark 4.1 in [37]) there exist positive constants $\textsf{C}_{\texttt {INV}}$ and ${\widehat{\textsf{C}}}_{\texttt {INV}}$ independent of the partition of ${\varOmega }$, such that

1.12 $$\begin{aligned} h\,\Vert \chi \Vert _1+h^{\frac{d}{2}}\,|\chi |_{\infty } \le \,\textsf{C}_{{\texttt {INV}}}\,\Vert \chi \Vert \quad \forall \,\chi \in {\textsf{S}_h}\end{aligned}$$

and

1.13 $$\begin{aligned} |\chi |_{\infty }\le \,{\widehat{\textsf{C}}}_{{\texttt {INV}}} \,\varphi _{\star }(d,h)\,\Vert \chi \Vert _1 \quad \forall \,\chi \in {\textsf{S}_h},\quad d\in \{2,3\}, \end{aligned}$$

where

1.14 $$\begin{aligned} \varphi _{\star }(d,h):=\left\{ \begin{aligned}&\,1,\hspace{71.13188pt}\,d=1\\&\,h^{\frac{2-d}{2}}\,|\ln (h)|^{\frac{d-1}{d}},\quad d\in \{2,3\} \end{aligned}\right. . \end{aligned}$$

Thus, combining (1.9), (1.10), (1.11) and (1.12) (see, e.g. Lemma 1 in [4], Section 3 in [17]), it follows that

1.15 $$\begin{aligned} |\textsf{R}_h{v}-v|_{\infty }\le \,\textsf{C}_{{\texttt {EP2}}}\,h^{2-\frac{d}{2}}\,\Vert v\Vert _2 \quad \forall \,v\in {\mathbb {H}}^2({\varOmega }). \end{aligned}$$

Let $N\in {\mathbb {N}}$. The time-discretization of the problem will be based on a uniform partition of [0, *T*] with nodes $(t_n)_{n=0}^{\scriptscriptstyle N}$ defined by $t_n:=n\,\tau$ for $n=0,\dots , N$, where $\tau :=\frac{T}{N}$ is the time-step. Also, given a set of functions $(\Upsilon ^n)_{n=0}^{\scriptscriptstyle N}$ corresponding to the nodes $(t_n)_{n=0}^{\scriptscriptstyle N}$, we introduce the discrete time-derivatives $\partial _{\tau }\Upsilon ^n:=\tfrac{\Upsilon ^{n}-\Upsilon ^{n-1}}{\tau }$ and the time-averages ${\mathcal {A}}_{\tau }\Upsilon ^n:= \tfrac{\Upsilon ^{n}+\Upsilon ^{n-1}}{2}$ for $n=1,\dots ,N$. Later, in the error estimation, we will often arrive at a recursive inequality of the form

1.16 $$\begin{aligned} q_{\scriptscriptstyle A}^{n}\le \,e^{{\mathfrak c}_{\star }\tau } \,q_{\scriptscriptstyle A}^{n-1}+q^n_{\scriptscriptstyle B},\quad n=1,\dots ,N, \end{aligned}$$

where ${\mathfrak c}_{\star }$, $(q_{\scriptscriptstyle A}^n)_{n=0}^{\scriptscriptstyle N}$ and $(q_{\scriptscriptstyle B}^n)_{n=1}^{\scriptscriptstyle N}$ are non-negative constants. Then, by induction, we can establish that

1.17 $$\begin{aligned} q_{\scriptscriptstyle A}^n\le \,e^{{\mathfrak c}_{\star }\,t_n} \,\left( q_{\scriptscriptstyle A}^0+\sum _{\ell =1}^nq_{\scriptscriptstyle B}^{\ell }\right) , \quad n=1,\dots ,N. \end{aligned}$$

### Linearly Implicit Finite Element Methods

We formulate below two linearly implicit finite element methods approximating the solution *u* to the problem (1.2)-(1.5) over the nodes $(t_n)_{n=0}^{\scriptscriptstyle N}$.

#### The Linearized Backward Euler Finite Element Method

Motivated by [12], we formulate below the Linearized Backward Euler Finite Element (LBEFE) method consisting of the following steps:

Step LBE0. Set

1.18 $$\begin{aligned} \textsf{U}_h^0:=\textsf{P}_h(u_0). \end{aligned}$$

Step LBE1. Compute $\textsf{U}_h^1\in {\textsf{S}_h}$ such that

1.19 $$\begin{aligned} \partial _{\tau }\textsf{U}_h^{1}+\textrm{i}\,{\varDelta }_h(\textsf{U}_h^1) =\textsf{P}_h\left[ \textrm{i}\,\left( V+\mu \,|u_0|^{\beta }\right) \,\textsf{U}_h^1 +f(t_1,\cdot )\right] . \end{aligned}$$

Step LBE2. For $n=2,\dots ,N$, find $\textsf{U}_h^{n}\in {\textsf{S}_h}$ such that

1.20 $$\begin{aligned} \partial _{\tau }\textsf{U}_h^n +\textrm{i}\,{\varDelta }_h(\textsf{U}_h^n) =\textsf{P}_h\left[ \textrm{i}\,\left( V+\mu \,|\textsf{U}_h^{n-1}|^{\beta }\right) \,\textsf{U}_h^n +f(t_n,\cdot )\right] . \end{aligned}$$

##### Remark 1.2

The linearization of *g*(*u*) at the core of the (LBEFE) method (see (1.20)) has the form $\textsf{g}_{\texttt {LBE}}(\textsf{U}_h^{n-1},\textsf{U}_h^n)$, where $\textsf{g}_{\texttt {LBE}}(z_1,z_2):=\textrm{i}\,\mu \,|z_1|^{\beta }\,z_2$ for $z_1,z_2\in {\mathbb {C}}$.

##### Remark 1.3

Taking into account that *V* is a real-valued function and using (1.6), we, easily, conclude that:

1.21 $$\begin{aligned} \Vert \textsf{U}_h^n\Vert ^2+\Vert \textsf{U}_h^n-\textsf{U}_h^{n-1}\Vert ^2=\Vert \textsf{U}_h^{n-1}\Vert ^2 +2\,\tau \,\textsf{Re}\left[ \left( f(t_n,\cdot ),\textsf{U}_h^n\right) \right] ,\quad n=1,\dots ,N, \end{aligned}$$

which reads that the (LBEFE) method is $L^2({\varOmega })$-dissipative, i.e. if $f\equiv 0$, then $\Vert \textsf{U}_h^n\Vert \le \Vert \textsf{U}_h^{n-1}\Vert$ for $n=1,\dots ,N$. Moreover, since the (LBEFE) method is linearly implicit, relation (1.21) yields the existence and uniqueness of the the corresponding numerical approximations.

#### The Linearized Crank–Nicolson Finite Element Method

In the sequel, we formulate the Linearized Crank–Nicolson Finite Element (LCNFE) method which is a finite element version of the finite difference method proposed in [30] and has the following algorithm:

Step LCNFE0. Set

1.22 $$\begin{aligned} \textsf{W}_h^0:=\textsf{R}_h(u_0). \end{aligned}$$

Step LCNFE1. Compute $\textsf{W}_h^{1}\in {\textsf{S}_h}$ such that

1.23 $$\begin{aligned} \partial _{\tau }\textsf{W}_h^1 +\textrm{i}\,{\varDelta }_h\left( {\mathcal {A}}_{\tau }\textsf{W}_h^1\right) =\textsf{P}_h\left[ \textrm{i}\,\left( V+\mu \,|u_0|^{\beta }\right) \,{\mathcal {A}}_{\tau }\textsf{W}_h^1 +\tfrac{f(t_1,\cdot )+f(t_{0},\cdot )}{2}\right] . \end{aligned}$$

Step LCNFE2. For $n=2,\dots ,N$, find $\textsf{W}_h^{n}\in {\textsf{S}_h}$ such that

1.24 $$\begin{aligned} \partial _{\tau }\textsf{W}_h^n +\textrm{i}\,{\varDelta }_h\left( {\mathcal {A}}_{\tau }\textsf{W}_h^n\right) =\textsf{P}_h\left[ \textrm{i}\,\left( V+\mu \,|\textsf{W}_h^{n-1}|^{\beta }\right) \,{\mathcal {A}}_{\tau }\textsf{W}_h^n +\tfrac{f(t_n,\cdot )+f(t_{n-1},\cdot )}{2}\right] . \end{aligned}$$

##### Remark 1.4

The linearization of *g*(*u*) at the core of the (LCNFE) method (see (1.24)) has the form $\textsf{g}_{\texttt {LCN}}(\textsf{W}_h^{n-1},\textsf{W}_h^n)$, where $\textsf{g}_{\texttt {LCN}}(z_1,z_2):=\textrm{i}\,\mu \,|z_1|^{\beta }\,\frac{z_1+z_2}{2} =\frac{1}{2}\,\left[ g(z_1)+g_{\texttt {LBE}}(z_1,z_2)\right]$ for $z_1,z_2\in {\mathbb {C}}$.

##### Remark 1.5

Since *V* is a real-valued function, we, easily, arrive at

1.25 $$\begin{aligned} \Vert \textsf{W}_h^n\Vert ^2=\Vert \textsf{W}_h^{n-1}\Vert ^2 +2\,\tau \,\textsf{Re}\left[ \left( \tfrac{f(t_n,\cdot )+f(t_{n-1},\cdot )}{2}, {\mathcal {A}}_{\tau }\textsf{W}_h^n\right) \right] ,\quad n=1,\dots ,N, \end{aligned}$$

which yields that the (LCNFE) method is $L^2({\varOmega })$-conservative, i.e. if $f\equiv 0$, then $\Vert \textsf{W}_h^n\Vert =\Vert \textsf{R}_h(u_0)\Vert$ for $n=0,\dots ,N$. Also, since the (LCNFE) method is linearly implicit, relation (1.25) yields that the corresponding numerical approximations are well-defined.

### Motivation and Main Results

The semilinear term of the nonlinear Schrödinger equation has, usually, the form $g(z)=\textrm{i}\,\phi (|z|^2)\,z$, where $\phi$ is a polynomial (see, e.g. [9, 14, 32]) or a $C^2([0,+\infty ))$ function (see, e.g. [4, 11, 12, 25, 39, 41, 43]). This is not the case here, because $\phi (s)=\mu \,s^{\frac{\beta }{2}}$ for $s\ge 0$, which is not differentiable at zero. The latter limited regularity of the non-linear part of the semilinear term makes the error estimation of a numerical method approximating the solution to the problem challenging, because the results obtained in the literature are based on the assumption that $\phi$ is once or twice differentiable, or, $\phi$ is locally Lipschitz on $[0,+\infty )$, or *g* has $C^2$ regularity (see, e.g., [1, 3, 4, 12, 16, 19, 21, 33, 35, 36, 39, 41–43]).

Here, our aim is to contribute to the numerical approximation of the nonlinear Schrödinger equation with a Schamel-type nonlinearity focusing on the development of a convergence analysis for the (LCNFE) and the (LBEFE) methods. Since both are linearly implicit, in order to avoid the enforcement of a CFL condition in their error analysis, it sounds natural to move along the lines of a technique proposed in [41] (see also [4, 26]), which consists of the formulation and the convergence analysis of the corresponding time discrete method. Unfortunately, this approach fails to reach the aforementioned target, because the linearizations $\textsf{g}_{\texttt {LBE}}$ and $\textsf{g}_{\texttt {LCN}}$ of *g*, used in the methods under investigation (see Remark 1.2 and Remark 1.4), are not locally Lipschitz on ${\mathbb {C}}^2$. In particular, it is not possible to show that the error between the fully discrete approximations and the elliptic projection of the time discrete approximations is a pure space error, i.e. that is independent of $\tau$. This situation led us to follow the traditional way of estimating the error between the fully discrete approximations and the elliptic projection of the solution to the problem, having in our mind that mesh conditions may not be avoided.

To investigate the convergence of the proposed methods, we introduce a modified version of them by (3.10)-(3.12) and (3.34)-(3.36), where the linearizations $\textsf{g}_{\texttt {LBE}}$ and $\textsf{g}_{\texttt {LCN}}$ are mollified properly by using a cut-off $\textsf{G}_{\lambda }$ of the identity function (see (3.1)) that depends on a positive parameter $\lambda$ (see, e.g. [43]). Choosing a large enough value ${\lambda _{\star }}$ of $\lambda$, we derive, without imposing any CFL condition, an optimal order $O(\tau +h^2)$ error estimate in the $L^2({\varOmega })$ norm for both modified methods, and an $O(\tau ^{\alpha }+h)$ error estimate in the $H^1({\varOmega })$ norm, where $\alpha =\tfrac{3}{4}$ for the modified (LBEFE) method and $\alpha =\frac{1}{2}$ for the modified (LCNFE) one. Assuming that *h* and $\tau$ are small enough, and using the available $H^1({\varOmega })$ error estimates, we are able to conclude, for both methods, that the modified approximations are bounded in the $L^{\infty }({\varOmega })$ norm by ${\lambda _{\star }}$, without further conditions when $d=1$, and under a rather mild mesh condition when $d=2$ or 3, which follows by imposing an inverse inequality relating the $L^{\infty }({\varOmega })$ and $H^1({\varOmega })$ norms (see (1.13)). Then, the mollifier acts as an identity (see (3.3)), resulting that the modified approximations are identical to those of the corresponding unmodified ones, and therefore their convergence properties are common. To the knowledge of the authors, it is the first time in the literature that error estimates for standard conforming finite element methods approximating the solution to (1.2) are provided.

We note that, for both methods, the order of the $H^1({\varOmega })$-convergence with respect to $\tau$ is suboptimal because the linearization terms $\textsf{g}_{\texttt {LBE}}$ and $\textsf{g}_{\texttt {LCN}}$ are not smooth enough, but we were not able to provide a numerical evidence of it (see Section 4) and thus we do not know if it is sharp or not. Moreover, we show that the order of convergence in the $H^1({\varOmega })$ norm of the (LBEFE) method is higher than that of the (LCNFE) method. The latter result follows because we are able to develop a stability argument for the (LBEFE) method which is based on (1.6) and limits the loss of the convergence rate due to the low regularity of the nonlinear term (cf. Theorem 1.1 in [27]).

The crucial step in our analysis is to overcome the loss of the local Lipschitz property by the linearizations $\textsf{g}_{\texttt {LBE}}$ and $\textsf{g}_{\texttt {LCN}}$. Since, each linearization follows from *g*(*u*) by approximating explicitly its nonlinear part and implicitly its linear one, a combination of the locally Lipschitz property (1.7) of *g* with a standard use of the Taylor formula allows us to arrive at an estimate of the consistency error of the corresponding time discretization (see Section 2). Finally, having the consistency error estimation as a compass, we succeed to provide convergence of the modified methods by handling efficiently the error between the linearized term and the corresponding linearized term in the consistency error equation which depends only on the exact solution to the problem.

Now, let us present some references related to the numerical approximation of the solution to the problem we consider here. The weak convergence of a Backward Euler finite difference scheme over ${\mathbb {R}}$ is discussed in [22], under the CFL condition $\tau =O(h^2)$. For the time-splitting method over the whole space ${\mathbb {R}}^d$ formulated in [23], a convergence analysis has been developed by the authors of [15], that concludes an $O(\tau ^{\frac{1}{2}})$ error estimate in the $L^2$-norm, when the initial data belong to $H^1$. In [8], when Dirichlet boundary conditions are imposed, a first order, time-splitting method combined by a sine pseudospectral space discretization is proposed, and a low regularity $O(\tau ^{\frac{1+\beta }{2}}+h^{1+\beta })$ error estimate in the $L^2$-norm is provided, which is based on an $\varepsilon$-regularization of *g* around the origin. In our opinion, due to the local Lipschitz property (1.7), no regularization of the nonlinear term is required and its use may affect the results of the error analysis. For the method proposed and analyzed in [8], when periodic boundary conditions are imposed, an $O(\tau +h^2)$ error estimate in the $L^2$-norm and an $O(\tau ^{\frac{1}{2}}+h)$ error estimate in the $H^1$-norm is shown in [6] under the CFL condition $\tau =O(h^2)$, and an $O(\tau +h^m)$ error estimate in the $L^2$-norm and an $O(\tau ^{\frac{1}{2}}+h^{m-1})$ in the $H^1$-norm is provided in [24], where *m* indicates the space regularity of the solution. Also, space-time finite element methods are proposed and numerically tested in [2], when periodic boundary conditions are imposed without providing an error analysis. Our work has been motivated by [30], where a first order in time and second order in space, linearized Crank–Nicolson finite difference method is formulated and computationally tested without providing an error analysis. The latter method is attractive because it conserves the $L^2$ norm in a discrete way, and several preliminary numerical tests on the problem at hand, indicated that a conservative method is more preferable than a non conservative one. Here, we, also, consider a linearization of the Backward Euler method (see [12]), even thought it is dissipative, in order to compare the methods both in theory and practice.

We close the introduction by giving a brief outline of the paper. In Section 2 we define and estimate the consistency error of the time discretization of our numerical methods. In Section 3 we investigate the convergence of the (LBEFE) and (LCNFE) methods by deriving a priori error estimates in the $L^2({\varOmega })$ and $H^1({\varOmega })$ norms. Finally, we expose results from numerical experiments in Section 4, and give a summary of our results in Section 5.

## Consistency Error of the Time-Discretization

Here, we define the consistency error of the time-discretization employed by the (LBEFE) and (LCNFE) methods, which then is estimated in the $L^2({\varOmega })$ norm by using Taylor expansions.

### Time-Discretization in the (LBEFE) Method

For $n=1,\dots , N$, the consistency error $\zeta _n\in L^2({\varOmega })$ of the time-discretization in the $(\textsf{LBEFE})$ method is defined by

2.1 $$\begin{aligned} \tfrac{u^n-u^{n-1}}{\tau }=\textrm{i}\,{\varDelta }u^{n} +\textrm{i}\,V\,u^{n} +\textrm{i}\,\mu \,|u^{n-1}|^{\beta }\,u^n+f^n+\zeta _n, \end{aligned}$$

where $u^m:=u(t_m,\cdot )$ for $m=0,\dots ,N$, and $f^m:=f(t_m,\cdot )$ for $m=1,\dots ,N$.

#### Proposition 2.1

Let us assume that $u\in C^2([0,T],L^2({\varOmega }))\cap C([0,T],{\mathbb {H}}^2({\varOmega }))$. Then, for the consistency errors $(\zeta _n)_{n=1}^{\scriptscriptstyle N}$ defined by (2.1), it holds that

2.2 $$\begin{aligned} \Vert \zeta _n\Vert ^2\le \,{\mathfrak c}_{\texttt {LBE}}(u) \,\tau \,\textsf{Q}_{\scriptscriptstyle {\texttt {LBE}}}^n(u), \quad n=1,\dots ,N, \end{aligned}$$

where $${\mathfrak c}_{\texttt {LBE}}(u):=2\,\max \big \{\frac{1}{3}, 2\,\mu ^2\, \max \limits _{\scriptscriptstyle [0,T]}|u|_{\infty }^{2\beta }\,( 1+16\,(1+\beta )^2\,)\big \}$$ and

$$\begin{aligned} \textsf{Q}_{\scriptscriptstyle {\texttt {LBE}}}^n(u):=\int _{t_{n-1}}^{t_n} \left( \Vert u_{t}(s,\cdot )\Vert ^2+\Vert u_{tt}(s,\cdot )\Vert ^2\right) \,\;ds. \end{aligned}$$

**Proof**

Let $n\in \{1,\dots ,N\}$. Subtracting (1.2) at time $t=t_{n}$ from (2.1) we get $\zeta _n=-\zeta _n^a+\zeta ^b_n$, where $\zeta _n^a:=\textrm{i}\,\mu \,|u^{n-1}|^{\beta }\,u^n-g(u^n)$ and $\zeta _n^b:=\partial _{\tau }u^n-u_t(t_{n},\cdot )$. Then, we use the arithmetic mean inequality and (1.7), to obtain

2.3 $$\begin{aligned} \Vert \zeta _n\Vert ^2\le \,2\,\left( \Vert \zeta _n^{a}\Vert ^2 +\Vert \zeta _n^{b}\Vert ^2\right) \end{aligned}$$

and

2.4 $$\begin{aligned} \begin{aligned} \Vert \zeta ^a_n\Vert ^2=&\,\Vert \textrm{i}\,\mu \,|u^{n-1}|^{\beta }\,(u^n-u^{n-1}) +g(u^{n-1})-g(u^{n})\Vert ^2\\ \le&\,2\,\mu ^2\,\max _{\scriptscriptstyle [0,T]}|u|_{\infty }^{2\beta } \,\Vert u^n-u^{n-1}\Vert ^2+ 2\,\Vert g(u^{n-1})-g(u^{n})\Vert ^2\\ \le&\,2\,\mu ^2\, \max \limits _{\scriptscriptstyle [0,T]}|u|_{\infty }^{2\beta }\,\left( 1+16\,(1+\beta )^2\,\right) \,\Vert u^n-u^{n-1}\Vert ^2.\\ \end{aligned} \end{aligned}$$

Also, applying the Taylor formula to expand $u^{n-1}$ around $t=t_{n}$, it follows that

$$\begin{aligned} \zeta ^b_n=\int _{t_{n-1}}^{t_n}\tfrac{t_{n-1}-s}{\tau }\,u_{tt}(s,\cdot )\;ds, \end{aligned}$$

which yields

2.5 $$\begin{aligned} \begin{aligned} \Vert \zeta _n^b\Vert ^2=&\,\int _{\scriptscriptstyle {\varOmega }}\Big |\int _{t_{n-1}}^{t_n} \tfrac{s-t_{n-1}}{\tau }\,u_{tt}(s,x)\;ds\Big |^2\;dx\\ \le&\,\int _{\scriptscriptstyle {\varOmega }}\left( \int _{t_{n-1}}^{t_n} |u_{tt}(s,x)|^2\;ds\right) \,\left( \int _{t_{n-1}}^{t_n} \tfrac{(s-t_{n-1})^2}{\tau ^2}\;ds\right) \;dx\\ \le&\,\tfrac{\tau }{3}\,\int _{t_{n-1}}^{t_n} \Vert u_{tt}(s,\cdot )\Vert ^2\;ds.\\ \end{aligned} \end{aligned}$$

Observing that

$$\begin{aligned} \begin{aligned} \Vert u^{n}-u^{n-1}\Vert ^2 \le&\, \int _{\scriptscriptstyle {\varOmega }}\left( \int _{t_{n-1}}^{t_n}\,|u_{t}(s,x)|\;ds\right) ^2\;dx\\ \le&\, \int _{\scriptscriptstyle {\varOmega }}\left( \int _{t_{n-1}}^{t_n}1\;dx\right) \left( \int _{t_{n-1}}^{t_n}\,|u_{t}(s,x)|^2\;ds\right) \;dx\\ \le&\,\tau \, \int _{t_{n-1}}^{t_n}\,\Vert u_{t}(s,\cdot )\Vert ^2\;ds,\\ \end{aligned} \end{aligned}$$

(2.2) follows, easily, in light of (2.3), (2.4) and (2.5).

### Time-Discretization in the (LCNFE) Method

For $n=1,\dots ,N$, the consistency error $\xi _n\in L^2({\varOmega })$ of time-discretization in the $(\mathsf LCNFE)$ method is specified by

2.6 $$\begin{aligned} \tfrac{u^n-u^{n-1}}{\tau } =\textrm{i}\,{\varDelta }\left( \tfrac{u^{n}+u^{n-1}}{2}\right) +\textrm{i}\,V\,\tfrac{u^{n}+u^{n-1}}{2} +\textrm{i}\,\mu \,|u^{n-1}|^{\beta }\,\tfrac{u^n+u^{n-1}}{2} +\tfrac{f^{n}+f^{n-1}}{2}+\xi _n, \end{aligned}$$

where $u^m:=u(t_m,\cdot )$ and $f^m:=f(t_m,\cdot )$ for $m=0,\dots ,N$.

#### Proposition 2.2

Let us assume that $u\in C^2([0,T],L^2({\varOmega }))\cap C([0,T],{\mathbb {H}}^2({\varOmega }))$, and let $(\xi _n)_{n=1}^{\scriptscriptstyle N}$ be the consistency errors defined by (2.6). Then, it holds that

2.7 $$\begin{aligned} \Vert \xi _n\Vert \le \,{\mathfrak c}_{\texttt {LCN}}(u)\,\textsf{Q}_{\scriptscriptstyle {\texttt {LCN}}}^n(u), \quad n=1,\dots ,N, \end{aligned}$$

where $${\mathfrak c}_{\texttt {LCN}}(u):=\max \big \{1, |\mu |\,\tfrac{5+4\,\beta }{2} \max \limits _{\scriptscriptstyle [0,T]}|u|_{\infty }^{\beta }\big \}$$ and $$\textsf{Q}_{\scriptscriptstyle {\texttt {LCN}}}^n(u):=\int _{t_{n-1}}^{t_n} (\Vert u_{t}(s,\cdot )\Vert +\Vert u_{tt}(s,\cdot )\Vert )\,\;ds$$.

**Proof**

Let $n\in \{1,\dots ,N\}$. We subtract the average of (1.2) at $t=t_{n}$ and $t=t_{n-1}$ from (2.6) to get

2.8 $$\begin{aligned} \xi _n=\xi _n^{a}+\xi _n^b-\xi _n^{c} \end{aligned}$$

where

$$\begin{aligned} \begin{aligned} \xi _n^{a}:=&\,\tfrac{u(t_n,\cdot )-u(t_{n-1},\cdot )}{\tau } -u_t(t_n-\tfrac{\tau }{2},\cdot ),\\ \xi _n^{b}:=&\,u_t(t_n-\tfrac{\tau }{2},\cdot ) -\tfrac{u_t(t_n,\cdot )+u_t(t_{n-1},\cdot )}{2},\\ \xi _n^{c}:=&\,\textrm{i}\,\mu \,|u^{n-1}|^{\beta }\,\tfrac{u^n+u^{n-1}}{2} -\tfrac{g(u^n)+g(u^{n-1})}{2}.\\ \end{aligned} \end{aligned}$$

Applying the Taylor formula we expand $u(t_n,\cdot )$, $u(t_{n-1},\cdot )$, $u_t(t_n,\cdot )$ and $u_t(t_{n-1},\cdot )$ around $t=t_{n}-\frac{\tau }{2}$, to obtain

$$\begin{aligned} \begin{aligned} \xi _n^{a}=\int _{t_{n}-\frac{\tau }{2}}^{t_n} u_{tt}(s,\cdot )\,\left( \tfrac{t_n-s}{\tau }\right) \;ds +\int _{t_{n-1}}^{t_{n}-\frac{\tau }{2}} u_{tt}(s,\cdot )\,\left( \tfrac{t_{n-1}-s}{\tau }\right) \;ds \end{aligned} \end{aligned}$$

and

$$\begin{aligned} \begin{aligned} \xi _n^{b}=-\tfrac{1}{2}\,\int _{t_{n}-\frac{\tau }{2}}^{t_n} u_{tt}(s,\cdot )\;ds +\tfrac{1}{2}\,\int _{t_{n-1}}^{t_{n}-\frac{\tau }{2}} u_{tt}(s,\cdot )\;ds, \end{aligned} \end{aligned}$$

which yield

2.9 $$\begin{aligned} \Vert \xi _n^{a}+\xi _n^{b}\Vert \le \,\int _{t_{n-1}}^{t_n}\Vert u_{tt}(s,\cdot )\Vert \;ds. \end{aligned}$$

Observing that

$$\begin{aligned} \begin{aligned} \xi _n^{c}=&\,\tfrac{1}{2}\,\left[ \, \textrm{i}\,\mu \,|u^{n-1}|^{\beta }\,u^n-g(u^n)\,\right] \\ =&\,\tfrac{1}{2}\,\left[ \,\textrm{i}\,\mu \,|u^{n-1}|^{\beta }\,(u^n-u^{n-1}) +g(u^{n-1})-g(u^n)\right] ,\\ \end{aligned} \end{aligned}$$

we use (1.7) to arrive at

2.10 $$\begin{aligned} \begin{aligned} \Vert \xi ^{c}_n\Vert =&\,\tfrac{1}{2}\,\left[ \, |\mu |\,|u^{n-1}|_{\infty }^{\beta }\,\Vert u^n-u^{n-1}\Vert +\Vert g(u^{n})-g(u^{n-1})\Vert \,\right] \\ \le&\,|\mu |\,\tfrac{5+4\,\beta }{2} \,\max \limits _{\scriptscriptstyle [0,T]}|u|_{\infty }^{\beta }\,\Vert u^n-u^{n-1}\Vert \\ \le&\,|\mu |\,\tfrac{5+4\,\beta }{2} \,\max \limits _{\scriptscriptstyle [0,T]}|u|_{\infty }^{\beta } \,\int _{t_{n-1}}^{t_n}\Vert u_t(s,\cdot )\Vert \;ds.\\ \end{aligned} \end{aligned}$$

Thus, (2.7) follows, easily, as a consequence of (2.8), (2.9) and (2.10).

## Convergence Analysis

In this section, we investigate the convergence in the $L^2({\varOmega })$ and $H^1({\varOmega })$ norms of the (LBEFE) and (LCNFE) methods. The basic ingredient of the analysis we develop is the construction and the error estimation of a modified version of the aforementioned methods, where the nonlinear term is mollified properly by a smooth cut-off function.

### An Auxiliary Smooth Cut-Off Function.

For $\lambda >0$, we define a complex-valued cut-off function $\textsf{G}_{\lambda }:{\mathbb {C}}\rightarrow {\mathbb {C}}$ by

3.1 $$\begin{aligned} \textsf{G}_{\lambda }(z):=\textsf{g}_{\lambda }(\textsf{Re}(z))+\textrm{i}\,\textsf{g}_{\lambda }(\textsf{Im}(z)) \quad \forall \,z\in {\mathbb {C}}, \end{aligned}$$

where (see, e.g. (3.1)-(3.2) in [29]) $\textsf{g}_{\lambda }\in C^1({\mathbb {R}},{\mathbb {R}})$ is a real valued cut-off function given by

$$\begin{aligned} \textsf{g}_{\lambda }(-s)=-\textsf{g}_{\lambda }(s) \quad \forall \,s\in {\mathbb {R}}\end{aligned}$$

and

3.2 $$\begin{aligned} \textsf{g}_{\lambda }(s):=\left\{ \begin{aligned}&s,\hspace{30.15985pt}\text{ if }\ \ s\in [0,\lambda ],\\&q_{\lambda }(s),\hspace{12.80365pt}\text{ if }\ \ s\in (\lambda ,2\lambda ],\\&2\,\lambda ,\hspace{23.33147pt}\text{ if }\ \ s> 2\lambda ,\\ \end{aligned} \right. \quad \forall \,s\ge 0, \end{aligned}$$

with $q_{\lambda }(s):=s+\lambda \,\left( 1-\tfrac{s}{\lambda }\right) ^2 \,\left( 2-\tfrac{s}{\lambda }\right)$ for $s\in [\lambda ,2\lambda ]$. Obviously it holds that

3.3 $$\begin{aligned} \textsf{G}_{\lambda }(z)=z\quad \text {if}\quad |z|\le \lambda , \end{aligned}$$

and it can be shown (see Lemma 3.1 in [29]) that

3.4 $$\begin{aligned} \sup _{\scriptscriptstyle {\mathbb {R}}}|\textsf{g}_{\lambda }|=2\,\lambda ,\quad \text {and}\quad \sup _{\scriptscriptstyle {\mathbb {R}}}|\textsf{g}_{\lambda }'|\le \,\tfrac{4}{3}, \end{aligned}$$

which yields

3.5 $$\begin{aligned} |\textsf{G}_{\lambda }(z)-\textsf{G}_{\lambda }(w)|\le \,\tfrac{4}{3}\,|z-w|\quad \forall \,z,w\in {\mathbb {C}}\end{aligned}$$

and

3.6 $$\begin{aligned} \sup _{\scriptscriptstyle {\mathbb {C}}}|\textsf{G}_{\lambda }|\le \,\sqrt{8}\,\,\lambda . \end{aligned}$$

Using $G_{\lambda }$ as a basic ingredient, we construct a smooth cut-off version $g_{\lambda }:{\mathbb {C}}\rightarrow {\mathbb {C}}$ of *g* by

3.7 $$\begin{aligned} g_{\lambda }(z):=g\left( \textsf{G}_{\lambda }(z)\right) \quad \forall \,z\in {\mathbb {C}}, \end{aligned}$$

which, in light of (3.3), (1.7), (3.5) and (3.6), has the following properties

3.8 $$\begin{aligned} g_{\lambda }(z)=g(z)\quad \text {if}\quad |z|\le \lambda , \end{aligned}$$

and

3.9 $$\begin{aligned} \Vert g_{\lambda }(v)-g_{\lambda }(w)\Vert \le \,\textsf{C}_{\texttt {L}}\,\lambda ^{\beta } \,\Vert v-w\Vert \quad \forall \,v,w\in L^2({\varOmega }), \quad \forall \,\lambda >0, \end{aligned}$$

where $$\textsf{C}_{\texttt {L}}:=\tfrac{8^{2+\frac{\beta }{2}}}{12} \,|\mu |\,(1+\beta )$$.

### Convergence of the (LBEFE) Method

To handle the nonlinearity of the problem, we introduce below a modified version of the (LBEFE) method.

Step $$\textsf{MLBEFE0}$$. Set

3.10 $$\begin{aligned} \textsf{U}_{\lambda ,h}^0:=\textsf{P}_h(u_0). \end{aligned}$$

Step $\textsf{MLBEFE1}$. Seek $\textsf{U}_{\lambda ,h}^1\in {\textsf{S}_h}$ such that

3.11 $$\begin{aligned} \partial _{\tau }\textsf{U}_{\lambda ,h}^1 +\textrm{i}\,{\varDelta }_h\textsf{U}_{\lambda ,h}^1 =\textsf{P}_h\left[ \,\textrm{i}\left( V+\mu \,|u_0|^{\beta }\right) \,\textsf{U}_{\lambda ,h}^1 +f(t_1,\cdot )\,\right] . \end{aligned}$$

Step $\textsf{MLBEFE2}$. For $n=2,\dots , N$, find $\textsf{U}_{\lambda ,h}^{n}\in {\textsf{S}_h}$ such that

3.12 $$\begin{aligned} \partial _{\tau }\textsf{U}_{\lambda ,h}^n +\textrm{i}\,{\varDelta }_h\textsf{U}_{\lambda ,h}^n =\textsf{P}_h\left[ \,\textrm{i}\left( V+\mu \,\big |\textsf{G}_{\lambda }(\textsf{U}_{\lambda ,h}^{n-1})\big |^{\beta }\right) \,\textsf{U}_{\lambda ,h}^{n}+f(t_n,\cdot )\,\right] . \end{aligned}$$

In the sequel, we establish the convergence of the (LBEFE) method in the $L^2({\varOmega })$ and $H^1({\varOmega })$ norms, by deriving analogous error estimates for its modified version.

#### Theorem 3.1

Let us assume that $u\in C^2([0,T],L^2({\varOmega }))\cap C^1([0,T],{\mathbb {H}}^2({\varOmega }))$, $f\in C([0,T],L^2({\varOmega }))$ and $V\in L^{\infty }({\varOmega })$, and let $\varphi _{\star }$ be the function defined by (1.14). Then, there exist positive constants $\textsf{C}_{\texttt {LBE}}^{\mathfrak 1}$, $\textsf{C}_{\texttt {LBE}}^{\mathfrak 2}$ and $\textsf{C}_{\texttt {LBE}}^{\mathfrak 3}$ such that: if

$$\begin{aligned} \textsf{C}_{\texttt {LBE}}^{\mathfrak 1}\,\left[ \varphi _{\star }(d,h) \,(\tau ^{\frac{3}{4}}+h)+h^{2-\frac{d}{2}}\right] \le \tfrac{{\lambda _{\star }}}{6}, \end{aligned}$$

then

3.13 $$\begin{aligned} \max _{1\le {m}\le {\scriptscriptstyle N}}\Vert \textsf{U}_h^m-u^m\Vert \le \, \textsf{C}_{\texttt {LBE}}^{\mathfrak 2}\,(\tau +h^{2}) \quad \text {and}\quad \max _{1\le {m}\le {\scriptscriptstyle N}}\Vert \nabla (\textsf{U}_h^m-u^m)\Vert \le \, \textsf{C}_{\texttt {LBE}}^{\mathfrak 3}\,(\tau ^{\frac{3}{4}}+h). \end{aligned}$$

**Proof**

For our convenience, we simplify the notation by setting $\eta ^m:=u^m-\textsf{R}_h(u^m)$, ${\vartheta }_h^m:=\textsf{R}_h(u^m)-\textsf{U}^m_{{\lambda _{\star }},h}$ and $\delta _h^m:=\Delta _h{\vartheta }_h^m$ for $m=1,\dots ,N$, where ${\lambda _{\star }}:=1+6\,\max \limits _{\scriptscriptstyle [0,T]}|u|_{\infty }$. Also, we will use the symbol *C* to denote a generic constant that is independent of $\tau$ and *h*, may change value from one place to the other and may depend on the solution *u* and its derivatives.

Part ${\mathfrak 1}$: To derive a set of equations for the discrete error functions $({\vartheta }_h^m)_{m=1}^{\scriptscriptstyle N}$, we use (2.1), (3.3), (3.10)-(3.12), to obtain

3.14 $$\begin{aligned} \left( {\vartheta }_h^1,\chi \right) +\textrm{i}\,\tau \,({\varDelta }_h{\vartheta }_h^1,\chi ) =\sum _{\ell =1}^3 (\textsf{A}_{\ell },\chi ) \quad \forall \,\chi \in {\textsf{S}_h}, \end{aligned}$$

where

$$\begin{aligned}\begin{gathered} \textsf{A}_1:=\left[ -1+\textrm{i}\,\tau \,\left( V +\mu \,|u_0|^{\beta }\right) \right] \,\eta ^1,\quad \textsf{A}_2:=\textrm{i}\,\tau \,\left( V+\mu \,|u_0|^{\beta }\right) \,{\vartheta }_h^1,\quad \textsf{A}_3:=\tau \,\zeta _1, \end{gathered}\end{aligned}$$

and

3.15 $$\begin{aligned} \left( {\vartheta }_h^{m}-{\vartheta }_h^{m-1},\chi \right) +\textrm{i}\,\tau \,({\varDelta }_h{\vartheta }_h^{m},\chi ) =\sum _{\ell =1}^7(\textsf{B}_{\ell }^m,\chi ) \quad \forall \,\chi \in {\textsf{S}_h},\quad m=2,\dots ,N, \end{aligned}$$

where

$$\begin{aligned} \begin{aligned} \textsf{B}_1^m:=&\,\textsf{R}_h(u^{m}-u^{m-1}) -(u^{m}-u^{m-1}),\\ \textsf{B}_2^m:=&\,\textrm{i}\,\tau \,\left[ V+\mu \, \big |\textsf{G}_{{\lambda _{\star }}}(\textsf{U}_{{\lambda _{\star }},h}^{m-1})\big |^{\beta }\right] \,\eta ^m,\\ \textsf{B}_3^m:=&\,\textrm{i}\,\tau \,\left[ V+\mu \, \big |\textsf{G}_{{\lambda _{\star }}}(\textsf{U}_{{\lambda _{\star }},h}^{m-1})\big |^{\beta }\right] \,{\vartheta }_h^m,\\ \textsf{B}_4^m:=&\, \,\tau \,\left[ g_{{\lambda _{\star }}}(u^{m-1}) -g_{{\lambda _{\star }}}(\textsf{U}_{{\lambda _{\star }},h}^{m-1})\right] ,\\ \textsf{B}_5^m:=&\,\textrm{i}\,\tau \,\mu \,\left[ \big |\textsf{G}_{{\lambda _{\star }}}(u^{m-1})\big |^{\beta } -\big |\textsf{G}_{{\lambda _{\star }}}(\textsf{U}_{{\lambda _{\star }},h}^{m-1})\big |^{\beta }\right] \, (u^{m}-u^{m-1}),\\ \textsf{B}_6^m:=&\,\textrm{i}\,\tau \,\mu \, \big |\textsf{G}_{{\lambda _{\star }}}(\textsf{U}_{{\lambda _{\star }},h}^{m-1})\big |^{\beta }\, \left[ \textsf{G}_{{\lambda _{\star }}}(\textsf{U}_{{\lambda _{\star }},h}^{m-1})-\textsf{G}_{{\lambda _{\star }}}(u^{m-1})\right] ,\\ \textsf{B}_7^m:=&\,\tau \,\zeta _m.\\ \end{aligned} \end{aligned}$$

Part ${\mathfrak 2}$: Setting $\chi ={\vartheta }_h^{1}$ in (3.14), observing that $\textsf{Re}[(\textsf{A}_2,{\vartheta }_h^1)]=0$, and using (1.8), the Cauchy-Schwarz inequality, (1.11) and the arithmetic mean inequality, we get

$$\begin{aligned} \begin{aligned} \Vert {\vartheta }_h^1\Vert ^2=&\,\textsf{Re}[(\textsf{A}_1+\textsf{A}_3,{\vartheta }_h^1)]\\ \le&\,C\,\left( \Vert \eta ^1\Vert +\tau \,\Vert \zeta ^1\Vert \right) \,\Vert {\vartheta }_h^1\Vert \\ \le&\,C\,\left( h^2\,\Vert u^1\Vert _2+\tau \,\Vert \zeta ^1\Vert \right) ^2 +\tfrac{1}{4}\,\Vert {\vartheta }_h^1\Vert ^2\\ \le&\,C\,\left( h^4+\tau ^2\,\Vert \zeta _1\Vert ^2 \right) +\tfrac{1}{4}\,\Vert {\vartheta }_h^1\Vert ^2,\\ \end{aligned} \end{aligned}$$

which, along with (2.2), yields

3.16 $$\begin{aligned} \Vert {\vartheta }_h^1\Vert ^2\le \,C\,\left[ \,h^4+\tau ^3\,\textsf{Q}_{\scriptscriptstyle {\texttt {LBE}}}^1(u)\,\right] . \end{aligned}$$

Also, setting $\chi =\delta _h^1$ in (3.14) and using (1.8), we obtain

$$\begin{aligned} \Vert \nabla {\vartheta }_h^1\Vert ^2 +\textrm{i}\,\tau \,\Vert \delta _h^1\Vert ^2 =\sum _{\ell =1}^3 (\textsf{A}_{\ell },\delta _h^1), \end{aligned}$$

which, along with the Cauchy-Schwarz inequality, (1.11) and the arithmetic mean inequality, yields

$$\begin{aligned} \begin{aligned} \Vert \delta _h^1\Vert ^2=&\,\tfrac{1}{\tau }\, \textsf{Im}[(\textsf{A}_1+\textsf{A}_2+\textsf{A}_3,\delta _h^1)]\\ \le&\,C\,\tfrac{1}{\tau }\,\left( \Vert \eta ^{1}\Vert +\tau \,\Vert {\vartheta }_h^1\Vert +\tau \,\Vert \zeta _1\Vert \right) \,\Vert \delta _h^1\Vert \\ \le&\,C\,\left( \tfrac{h^2}{\tau } +\Vert {\vartheta }_h^1\Vert +\Vert \zeta _1\Vert \right) \,\Vert \delta _h^1\Vert \\ \le&\,C\,\left( \tfrac{h^4}{\tau ^2}+\Vert \zeta _1\Vert ^2 +\Vert {\vartheta }_h^1\Vert ^2\right) +\tfrac{1}{2}\,\Vert \delta _h^1\Vert ^2. \end{aligned} \end{aligned}$$

Finally, by combining the inequality above with (3.16) and (2.2), we arrive at

3.17 $$\begin{aligned} \Vert \delta _h^1\Vert ^2\le \,C\,\left[ \,\tfrac{h^4}{\tau ^2}+h^4 +\tau \,\textsf{Q}_{\scriptscriptstyle {\texttt {LBE}}}^1(u)\,\right] . \end{aligned}$$

Part ${\mathfrak 3}$: Let $m\in \{2,\dots ,N\}$. Setting $\chi ={\vartheta }_h^{m}$ in (3.15), taking real parts and using (1.6) and (1.8), we get

3.18 $$\begin{aligned} \Vert {\vartheta }_h^m\Vert ^2-\Vert {\vartheta }_h^{m-1}\Vert ^2+\Vert {\vartheta }_h^m-{\vartheta }_h^{m-1}\Vert ^2= 2\,\sum _{\ell =1}^7\textsf{Re}\left[ (\textsf{B}_{\ell }^m,{\vartheta }_h^m)\right] . \end{aligned}$$

Using the Cauchy-Schwarz inequality, (1.11), (3.6), (3.9), (3.5), (2.2), and the arithmetic mean inequality, we obtain the following bounds

3.19 $$\begin{aligned} \begin{aligned} \big |(\textsf{B}_1^m,{\vartheta }_h^m)\big | \le&\,C\,h^2\,\big \Vert u^{m}-u^{m-1}\big \Vert _2 \,\Vert {\vartheta }_h^m\Vert \\ \le&\,C\,h^2\,\Vert u^{m}-u^{m-1}\Vert _2 \,\left( \Vert {\vartheta }_h^m-{\vartheta }_h^{m-1}\Vert +\Vert {\vartheta }_h^{m-1}\Vert \right) \\ \le&\,C\,h^4\,\Vert u^m-u^{m-1}\Vert _2^2 +\tfrac{1}{10}\,\Vert {\vartheta }_h^m-{\vartheta }_h^{m-1}\Vert ^2\\&\quad +C\,h^4\tau ^{-1}\,\Vert u^m-u^{m-1}\Vert _2^2 +\tau \,\Vert {\vartheta }_h^{m-1}\Vert ^2\\ \le&\,C\,h^4\,\tau ^{-1}\Vert u^m-u^{m-1}\Vert _2^2 +\tfrac{1}{10}\,\Vert {\vartheta }_h^m-{\vartheta }_h^{m-1}\Vert ^2 +\tau \,\Vert {\vartheta }_h^{m-1}\Vert ^2\\ \le&\,\tfrac{1}{10}\,\Vert {\vartheta }_h^m-{\vartheta }_h^{m-1}\Vert ^2 +\tau \,\Vert {\vartheta }_h^{m-1}\Vert ^2 +C\,h^4\,\int _{t_{m-1}}^{t_m} \Vert u_t(s,\cdot )\Vert _2^2\,ds,\\ \end{aligned} \end{aligned}$$

3.20 $$\begin{aligned} \begin{aligned} \big |(\textsf{B}_2^m+\textsf{B}_4^m+\textsf{B}_6^m,{\vartheta }_h^m)\big | \le&\,C\,\tau \,\left( \Vert \eta ^m\Vert +\Vert u^{m-1}-\textsf{U}_{{\lambda _{\star }},h}^{m-1}\Vert \right) \,\Vert {\vartheta }_h^m\Vert \\ \le&\,C\,\tau \,\left( \Vert \eta ^m\Vert +\Vert \eta ^{m-1}\Vert +\Vert {\vartheta }_h^{m-1}\Vert \right) \,\Vert {\vartheta }_h^m\Vert \\ \le&\,C\,\tau \,\left[ h^2\,(\Vert u^m\Vert _2+\Vert u^{m-1}\Vert _2) +\Vert {\vartheta }_h^{m-1}\Vert \right] \,\Vert {\vartheta }_h^m\Vert \\ \le&\,C\,\tau \,(h^2+\Vert {\vartheta }_h^{m-1}\Vert ) \,\Vert {\vartheta }_h^m\Vert \\ \le&\,C\,\tau \,\left( h^2+\Vert {\vartheta }_h^{m-1}\Vert \right) \,\left( \Vert {\vartheta }_h^m-{\vartheta }_h^{m-1}\Vert +\Vert {\vartheta }_h^{m-1}\Vert \right) \\ \le&\,C\,\tau \,\left( h^2+\Vert {\vartheta }_h^{m-1}\Vert \right) \,\Vert {\vartheta }_h^m-{\vartheta }_h^{m-1}\Vert +C\,\tau \,\left( h^2+\Vert {\vartheta }_h^{m-1}\Vert \right) ^2\\ \le&\,\tfrac{1}{10}\,\Vert {\vartheta }_h^m-{\vartheta }_h^{m-1}\Vert ^2 +C\,\tau \,\left( h^4+\Vert {\vartheta }_h^{m-1}\Vert ^2\right) \\ \end{aligned} \end{aligned}$$

and

3.21 $$\begin{aligned} \begin{aligned} \big |(\textsf{B}_5^m+\textsf{B}_7^m,{\vartheta }_h^m)\big | \le&\,C\,\tau \,\left( \Vert u^{m}-u^{m-1}\Vert +\Vert \zeta _m\Vert \right) \, \,\left( \Vert {\vartheta }_h^m-{\vartheta }_h^{m-1}\Vert +\Vert {\vartheta }_h^{m-1}\Vert \right) \\ \le&\,C\,\tau \,\left( \Vert u^{m}-u^{m-1}\Vert +\Vert \zeta _m\Vert \right) \, \,\Vert {\vartheta }_h^m-{\vartheta }_h^{m-1}\Vert \\&\hspace{28.45274pt}+C\,\tau \,\left( \Vert u^{m}-u^{m-1}\Vert +\Vert \zeta _m\Vert \right) \,\Vert {\vartheta }_h^{m-1}\Vert \\ \le&\,C\,\tau \,\left( \Vert u^{m}-u^{m-1}\Vert ^2+\Vert \zeta _m\Vert ^2\right) \, +\tfrac{1}{10}\,\Vert {\vartheta }_h^m-{\vartheta }_h^{m-1}\Vert ^2 +\tau \,\Vert {\vartheta }_h^{m-1}\Vert ^2\\ \le&\,C\,\tau \,\left( \tau \,\int _{t_{m-1}}^{t_m}\Vert u_t(s,\cdot )\Vert ^2\,ds +\Vert \zeta _m\Vert ^2\right) \, +\tfrac{1}{10}\,\Vert {\vartheta }_h^m-{\vartheta }_h^{m-1}\Vert ^2 +\tau \,\Vert {\vartheta }_h^{m-1}\Vert ^2\\ \le&\,C\,\tau ^2\,\textsf{Q}_{\scriptscriptstyle {\texttt {LBE}}}^m(u) +\tfrac{1}{10}\,\Vert {\vartheta }_h^m-{\vartheta }_h^{m-1}\Vert ^2 +\tau \,\Vert {\vartheta }_h^{m-1}\Vert ^2.\\ \end{aligned} \end{aligned}$$

In light of $\textsf{Re}(\textsf{B}_3^m,{\vartheta }_h^m)=0$, (3.18), (3.19), (3.20) and (3.21), we arrive at

3.22 $$\begin{aligned} \begin{aligned} \Vert {\vartheta }_h^{m}\Vert ^2+\tfrac{7}{10} \,\Vert {\vartheta }_h^{m}-{\vartheta }_h^{m-1}\Vert ^2\le&\, (1+C\,\tau )\,\Vert {\vartheta }_h^{m-1}\Vert ^2+C\,Q_{\scriptscriptstyle {\texttt {LBE}}}^m\\ \le&\,e^{C\,\tau }\,\Vert {\vartheta }_h^{m-1}\Vert ^2 +C\,Q_{\scriptscriptstyle {\texttt {LBE}}}^m,\quad m=2,\dots ,N,\\ \end{aligned} \end{aligned}$$

where

$$\begin{aligned} Q_{\scriptscriptstyle {\texttt {LBE}}}^m:=\tau \,h^4+\tau ^2\,\textsf{Q}_{\scriptscriptstyle {\texttt {LBE}}}^m(u) +h^4\,\int _{t_{m-1}}^{t_m}\Vert u_t(s,\cdot )\Vert _2^2\,ds. \end{aligned}$$

By applying a standard discrete Gronwall argument on (3.22) (see (1.16)-(1.17)) and then using (3.16), we conclude that

3.23 $$\begin{aligned} \begin{aligned} \max _{1\le {m}\le {\scriptscriptstyle N}}\Vert {\vartheta }_h^{m}\Vert ^2 \le&\,C\,\left( \Vert {\vartheta }_h^1\Vert ^2+\sum _{\ell =2}^{\scriptscriptstyle N}Q_{\scriptscriptstyle {\texttt {LBE}}}^{\ell }\right) \\ \le&\,C\,(\tau ^2+h^4).\\ \end{aligned} \end{aligned}$$

Part ${\mathfrak 4}$: Let $m\in \{2,\dots ,N\}$. Setting $\chi =\delta _h^m-\delta _h^{m-1}$ in (3.15), we have

$$\begin{aligned} \Vert \nabla ({\vartheta }_h^{m}-{\vartheta }_h^{m-1})\Vert ^2 +\textrm{i}\,\tau \,\left( \delta _h^{m},\delta _h^m-\delta _h^{m-1}\right) =\sum _{\ell =1}^7\left( \textsf{B}_{\ell }^m,\delta _h^m-\delta _h^{m-1}\right) , \end{aligned}$$

which, along with (1.6), yields

3.24 $$\begin{aligned} \Vert \delta _h^m\Vert ^2-\Vert \delta _h^{m-1}\Vert ^2 +\Vert \delta _h^m-\delta _h^{m-1}\Vert ^2=\tfrac{2}{\tau }\, \sum _{\ell =1}^7\textsf{Im}\left[ \left( \textsf{B}_{\ell }^m, \delta _h^m-\delta _h^{m-1}\right) \right] . \end{aligned}$$

Now, we use the Cauchy-Schwarz inequality, (1.11), (3.9), (3.5), (3.6), (2.2), (3.23) and the arithmetic mean inequality, to get

3.25 $$\begin{aligned} \begin{aligned} \tfrac{2}{\tau }\,\big |(\textsf{B}_2^m+\textsf{B}_3^m +\textsf{B}_4^m+\textsf{B}_6^m,\delta _h^m-\delta _h^{m-1})\big | \le&\,C\,\left( \Vert \eta ^m\Vert +\Vert {\vartheta }_h^{m}\Vert +\Vert \eta ^{m-1}\Vert +\Vert {\vartheta }_h^{m-1}\Vert \right) \,\Vert \delta _h^m-\delta _h^{m-1}\Vert \\ \le&\,C\,\left( h^2+\Vert {\vartheta }_h^{m-1}\Vert +\Vert {\vartheta }_h^{m}\Vert \right) \,\Vert \delta _h^m-\delta _h^{m-1}\Vert \\ \le&\,C\,\left( h^4+\Vert {\vartheta }_h^{m-1}\Vert ^2+\Vert {\vartheta }_h^{m}\Vert ^2\right) +\tfrac{1}{5}\,\Vert \delta _h^m-\delta _h^{m-1}\Vert ^2\\ \le&\,C\,\left( \tau ^2+h^4\right) +\tfrac{1}{5}\,\Vert \delta _h^m-\delta _h^{m-1}\Vert ^2,\\ \end{aligned} \end{aligned}$$

3.26 $$\begin{aligned} \begin{aligned} \tfrac{2}{\tau }\, \big |(\textsf{B}_5^m+\textsf{B}_7^m,\delta _h^m-\delta _h^{m-1})\big | \le&\,C\,\left( \Vert u^{m}-u^{m-1}\Vert +\Vert \zeta _m\Vert \right) \,\Vert \delta _h^m-\delta _h^{m-1}\Vert \\ \le&\,C\, \left( \Vert u^{m}-u^{m-1}\Vert ^2+\Vert \zeta _m\Vert ^2\right) +\tfrac{1}{5}\,\Vert \delta _h^m-\delta _h^{m-1}\Vert ^2\\ \le&\,C\,\tau \,\textsf{Q}_{\scriptscriptstyle {\texttt {LBE}}}^m(u) +\tfrac{1}{5}\,\Vert \delta _h^m-\delta _h^{m-1}\Vert ^2\\ \end{aligned} \end{aligned}$$

and

3.27 $$\begin{aligned} \begin{aligned} \tfrac{2}{\tau }\,\big |(\textsf{B}_1^m,\delta _h^m-\delta _h^{m-1})\big | \le&\,C\,\tfrac{h^2}{\tau }\,\big \Vert u^{m}-u^{m-1}\big \Vert _2 \,\Vert \delta _h^m-\delta _h^{m-1}\Vert \\ \le&\,C\,\tfrac{h^4}{\tau ^2}\, \Vert u^{m}-u^{m-1}\Vert ^2_2 +\tfrac{1}{5}\,\Vert \delta _h^m-\delta _h^{m-1}\Vert ^2\\ \le&\,\tfrac{1}{5}\,\Vert \delta _h^m-\delta _h^{m-1}\Vert ^2 +C\,\tfrac{h^4}{\tau }\, \int _{t_{m-1}}^{t_m}\Vert u_t(s,\cdot )\Vert _2^2\,ds.\\ \end{aligned} \end{aligned}$$

In light of (3.24), (3.25), (3.26) and (3.27), we obtain

3.28 $$\begin{aligned} \begin{aligned} \Vert \delta _h^m\Vert ^2-\Vert \delta _h^{m-1}\Vert ^2\le&\,C\,\left[ \tau ^2+h^4 +\tau \,\textsf{Q}_{\scriptscriptstyle {\texttt {LBE}}}^m(u) +\tfrac{h^4}{\tau }\,\int _{t_{m-1}}^{t_m} \Vert u_t(s,\cdot )\Vert _2\,ds\right] ,\quad m=2,\dots ,N,\\ \end{aligned} \end{aligned}$$

which, after summing with respect to *m* and using (3.17), yields

3.29 $$\begin{aligned} \begin{aligned} \max _{1\le {m}\le {\scriptscriptstyle N}}\Vert \delta _h^m\Vert ^2\le&\,\Vert \delta _h^1\Vert ^2 +C\,\left( \tau +\tfrac{h^4}{\tau }\right) \\ \le&\,C\,\left( \tau +\tfrac{h^4}{\tau ^2}\right) .\\ \end{aligned} \end{aligned}$$

Thus, (1.8), (3.29) and (3.23) yield

3.30 $$\begin{aligned} \begin{aligned} \max _{1\le {m}\le {\scriptscriptstyle N}}\Vert \nabla {\vartheta }_h^m\Vert =&\,\max _{1\le {m}\le {\scriptscriptstyle N}}\big |(\delta _h^m,{\vartheta }_h^m)\big |^{\frac{1}{2}}\\ \le&\,\max _{1\le {m}\le {\scriptscriptstyle N}}\left( \Vert \delta _h^m\Vert \,\Vert {\vartheta }_h^m\Vert \right) ^{\frac{1}{2}}\\ \le&\,C\,\left[ \left( \tau ^{\frac{1}{2}}+\tfrac{h^2}{\tau }\right) \,(\tau +h^2)\right] ^{\frac{1}{2}}\\ \le&\,C\,\left[ \tau ^{\frac{3}{2}}+(\tau ^{\frac{1}{2}}+1)\,h^2 +\left( \tfrac{h^2}{\tau }\right) \,h^2\right] ^{\frac{1}{2}}\\ \le&\,C\,\left[ \tau ^{\frac{3}{4}}+h\,\left( 1+\tfrac{h^2}{\tau } \right) ^{\frac{1}{2}}\right] .\\ \end{aligned} \end{aligned}$$

Also, using (1.12) and (3.23) it follows that

3.31 $$\begin{aligned} \max _{1\le {m}\le {\scriptscriptstyle N}}\Vert \nabla {\vartheta }_h^{m}\Vert \le \, C\,\left( h+\tfrac{\tau }{h}\right) . \end{aligned}$$

Finally, combining (3.31) and (3.30) (cf. [20]), we conclude that

3.32 $$\begin{aligned} \max _{1\le {m}\le {\scriptscriptstyle N}}\Vert \nabla {\vartheta }_h^{m}\Vert \le \,C\,\left\{ \begin{aligned}&\,2\,h,\hspace{42.67912pt}\text {if}\quad \sqrt{\tau }\le h\\&\,\tau ^{\frac{3}{4}}+h\,\sqrt{2}, \quad \text {if}\quad h\le \sqrt{\tau }\\ \end{aligned}\right. \quad \le \,C\,(\tau ^{\frac{3}{4}}+h). \end{aligned}$$

Part ${\mathfrak 5}$: Now, we use (1.15), (1.13) and (3.32), to get

3.33 $$\begin{aligned} \begin{aligned} |\textsf{U}^{m}_{{\lambda _{\star }},h}|_{\infty }\le&\, |{\vartheta }_h^{m}|_{\infty } +|\textsf{R}_h(u^m)-u^m|_{\infty } +|u^{m}|_{\infty }\\ \le&\,{\widehat{\textsf{C}}}_{\texttt {INV}}\,\varphi _{\star }(d,h) \,\Vert {\vartheta }_h^m\Vert _1 +\textsf{C}_{{\texttt {EP2}}}\,h^{2-\frac{d}{2}}\,\Vert u^m\Vert _2 +\tfrac{{\lambda _{\star }}}{6}\\ \le&\,C\,\varphi _{\star }(d,h) \,(\tau ^{\frac{3}{4}}+h) +\textsf{C}_{{\texttt {EP2}}}\,h^{2-\frac{d}{2}}\,\Vert u^m\Vert _2 +\tfrac{{\lambda _{\star }}}{6}\\ \le&\,\textsf{C}_{\texttt {LBE}}\,\left[ \varphi _{\star }(d,h)\, (\tau ^{\frac{3}{4}}+h)+h^{2-\frac{d}{2}}\right] +\tfrac{{\lambda _{\star }}}{6}, \quad m=1,\dots ,N.\\ \end{aligned} \end{aligned}$$

Requiring $\textsf{C}_{\texttt {LBE}}\,\left[ \varphi _{\star }(d,h)\, (\tau ^{\frac{3}{4}}+h)+h^{2-\frac{d}{2}}\right] \le \tfrac{{\lambda _{\star }}}{6}$, (3.33) yields that $\max \limits _{1\le {m}\le {\scriptscriptstyle N}} |\textsf{U}_{{\lambda _{\star }},h}^m|_{\infty } \le \,\tfrac{2\,{\lambda _{\star }}}{6}<{\lambda _{\star }}$, which, in light of (3.3), enforces that $\textsf{G}_{{\lambda _{\star }}}(\textsf{U}^m_{{\lambda _{\star }},h})=\textsf{U}^m_{{\lambda _{\star }},h}$ for $m=1,\dots ,N$. Combining the latter equality, along with (1.18)-(1.20) and (3.10)-(3.12), we arrive at $\textsf{U}^m_{{\lambda _{\star }},h}=\textsf{U}_h^m$ for $m=1,\dots ,N$, and hence (3.13) follows as a direct outcome of (3.23), (3.32) and (1.11).

### Convergence of the (LCNFE) Method

Proceeding in a similar manner, we consider a modified version of the (LCNFE) method, which is as follows.

Step $$\textsf{MLCNFE0}$$. Set

3.34 $$\begin{aligned} \textsf{W}_{\lambda ,h}^0:=\textsf{R}_h(u_0). \end{aligned}$$

Step $\textsf{MLCNFE1}$. Compute $\textsf{W}_{\lambda ,h}^1\in {\textsf{S}_h}$ such that

3.35 $$\begin{aligned} \partial _{\tau }\textsf{W}_{\lambda ,h}^1 +\textrm{i}\,{\varDelta }_h\left( {\mathcal {A}}_{\tau }\textsf{W}_{\lambda ,h}^1\right) =\textsf{P}_h\left[ \,\textrm{i}\,(V+\mu \,|u_0|^{\beta })\,{\mathcal {A}}_{\tau }\textsf{W}_{\lambda ,h}^1 +{\mathcal {A}}_{\tau }f^1\,\right] . \end{aligned}$$

Step $\textsf{MLCNFE2}$. For $n=2,\dots , N$, find $\textsf{W}_{\lambda ,h}^{n}\in {\textsf{S}_h}$ such that

3.36 $$\begin{aligned} \partial _{\tau }\textsf{W}_{\lambda ,h}^n +\textrm{i}\,{\varDelta }_h\left( {\mathcal {A}}_{\tau }\textsf{W}_{\lambda ,h}^n\right) =\textsf{P}_h\left[ \textrm{i}\,\left( V +\mu \,\big |\textsf{G}_{\lambda }(\textsf{W}_{\lambda ,h}^{n-1})\big |^{\beta }\right) \,{\mathcal {A}}_{\tau }\textsf{W}_{\lambda ,h}^n +{\mathcal {A}}_{\tau }f^n\right] . \end{aligned}$$

We provide convergence of the (LCNFE) method in the $L^2({\varOmega })$ and $H^1({\varOmega })$ norms, by investigating the convergence properties of its modified version.

#### Theorem 3.2

Let us assume that $u\in C^2([0,T],L^2({\varOmega }))\cap C^1([0,T],{\mathbb {H}}^2({\varOmega }))$, $V\in L^{\infty }({\varOmega })$ and $f\in C([0,T],L^2({\varOmega }))$, and let $\varphi _{\star }$ be the function defined in (1.14). Then, there exist positive constants $\textsf{C}_{\texttt {LCN}}^{\mathfrak 1}$, $\textsf{C}_{\texttt {LCN}}^{\mathfrak 2}$ and $\textsf{C}_{\texttt {LCN}}^{\mathfrak 3}$ such that: if

3.37 $$\begin{aligned} \textsf{C}_{\texttt {LCN}}^{\mathfrak 1}\,\left[ \varphi _{\star }(d,h)\,(\tau ^{\frac{1}{2}}+h)+h^{2-\frac{d}{2}}\right] \le \tfrac{{\lambda _{\star }}}{6}, \end{aligned}$$

then

3.38 $$\begin{aligned} \max _{1\le {m}\le {\scriptscriptstyle N}}\Vert \textsf{W}_h^m-u^m\Vert \le \, \textsf{C}_{\texttt {LCN}}^{\mathfrak 2}\,(\tau +h^{2}) \quad \text {and}\quad \max _{1\le {m}\le {\scriptscriptstyle N}}\Vert \nabla (\textsf{W}_h^m-u^m)\Vert \le \, \textsf{C}_{\texttt {LCN}}^{\mathfrak 3}\,(\tau ^{\frac{1}{2}}+h). \end{aligned}$$

#### Proof

For our convenience, we simplify the notation by setting $\eta ^m:=u^m-\textsf{R}_h(u^m)$ and $\theta _h^m:=\textsf{R}_h(u^m)-\textsf{W}_{{\lambda _{\star }},h}^m$ for $m=0,\dots ,N$, where ${\lambda _{\star }}:=1+6\,\max \limits _{\scriptscriptstyle [0,T]}|u|_{\infty }$. Also, we will use the symbol *C* to denote a generic constant that is independent of $\tau$ and *h*, may change value from one place to the other and may depend on the solution *u* and its derivatives.

Part I: In order to obtain a set of equations fulfilled by the error quantities $(\theta _h^m)_{m=1}^{\scriptscriptstyle N}$, we use (2.6), (3.3), (3.34)-(3.36), to conclude

3.39 $$\begin{aligned} (\theta _h^1,\chi )+\textrm{i}\,\tfrac{\tau }{2}\,(\nabla \theta _h^1,\nabla \chi ) =\tau \,\sum _{\ell =1}^4\big ({\widehat{\textsf{A}}}_{\ell },\chi \big ) \quad \forall \,\chi \in {\textsf{S}_h}, \end{aligned}$$

where

$$\begin{aligned} {\widehat{\textsf{A}}}_1:=\textsf{R}_h\left( \partial _{\tau }u^1\right) -\partial _{\tau }u^1,\quad {\widehat{\textsf{A}}}_2:=\tfrac{\textrm{i}}{2} \,\left( V+\mu \,|u_0|^{\beta }\right) \,\eta ^1,\quad {\widehat{\textsf{A}}}_3:=\tfrac{\textrm{i}}{2} \,\left( V+\mu \,|u_0|^{\beta }\right) \,\theta _h^1,\quad {\widehat{\textsf{A}}}_4:=\xi _1, \end{aligned}$$

and

3.40 $$\begin{aligned} (\theta _h^{m}-\theta _h^{m-1},\chi ) +\textrm{i}\,\tau \,\left( \nabla ({\mathcal {A}}_{\tau }\theta _h^{m}),\nabla \chi \right) =\tau \,\sum _{\ell =1}^5\big ({\widehat{\textsf{B}}}_{\ell }^m,\chi \big ) \quad \forall \chi \in {\textsf{S}_h}, \quad m=2,\dots ,N, \end{aligned}$$

where

$$\begin{aligned} {\widehat{\textsf{B}}}_1^m:=\textsf{R}_h\left( \partial _{\tau }u^{m}\right) -\partial _{\tau }u^m,\quad {\widehat{\textsf{B}}}_2^m:=\textrm{i}\,V\,{\mathcal {A}}_{\tau }\eta ^{m},\quad {\widehat{\textsf{B}}}_3^m:=\textrm{i}\,V\,{\mathcal {A}}_{\tau }\theta _h^{m},\quad {\widehat{\textsf{B}}}_4^m:=\xi _m \end{aligned}$$

and

$$\begin{aligned} {\widehat{\textsf{B}}}_5^m:=\textrm{i}\,\mu \,\left[ \, \big |\textsf{G}_{{\lambda _{\star }}}(u^{m-1})\big |^{\beta }\, {\mathcal {A}}_{\tau }u^m-\big |\textsf{G}_{{\lambda _{\star }}}(\textsf{W}_{{\lambda _{\star }},h}^{m-1})\big |^{\beta } \,{\mathcal {A}}_{\tau }\textsf{W}_{{\lambda _{\star }},h}^{m}\,\right] . \end{aligned}$$

By adding and subtracting appropriate terms, we rewrite ${\widehat{\textsf{B}}}_5^m$ in a more convenient form as follows

3.41 $$\begin{aligned} {\widehat{\textsf{B}}}_5^m=\sum _{\ell =1}^5{\widehat{\textsf{B}}}_{5,\ell }^m, \quad m=2,\dots ,N, \end{aligned}$$

where

$$\begin{aligned} \begin{aligned} {\widehat{\textsf{B}}}_{5,1}^m:=&\,\textrm{i}\,\mu \,\left[ \big |\textsf{G}_{{\lambda _{\star }}}(u^{m-1})\big |^{\beta } -\big |\textsf{G}_{{\lambda _{\star }}}(\textsf{W}_{{\lambda _{\star }},h}^{m-1})\big |^{\beta }\right] \, ({\mathcal {A}}_{\tau }u^{m}-u^{m-1}),\\ {\widehat{\textsf{B}}}_{5,2}^m:=&\,g_{{\lambda _{\star }}}(u^{m-1}) -g_{{\lambda _{\star }}}(\textsf{W}_{{\lambda _{\star }},h}^{m-1}),\\ {\widehat{\textsf{B}}}_{5,3}^m:=&\,\textrm{i}\,\mu \, \big |\textsf{G}_{{\lambda _{\star }}}(\textsf{W}_{{\lambda _{\star }},h}^{m-1})\big |^{\beta }\, \left[ \textsf{G}_{{\lambda _{\star }}}(\textsf{W}_{{\lambda _{\star }},h}^{m-1})-\textsf{G}_{{\lambda _{\star }}}(u^{m-1})\right] ,\\ {\widehat{\textsf{B}}}_{5,4}^m:=&\,\textrm{i}\,\mu \, \big |\textsf{G}_{{\lambda _{\star }}}(\textsf{W}_{{\lambda _{\star }},h}^{m-1})\big |^{\beta } \,{\mathcal {A}}_{\tau }\eta ^{m},\\ {\widehat{\textsf{B}}}_{5,5}^m:=&\,\textrm{i}\,\mu \, \big |\textsf{G}_{{\lambda _{\star }}}(\textsf{W}_{{\lambda _{\star }},h}^{m-1})\big |^{\beta } \,{\mathcal {A}}_{\tau }\theta _h^{m}.\\ \end{aligned} \end{aligned}$$

Part II: Our next step is to provide some basic bounds for the terms in the right-hand side of (3.39) and (3.40). First, we use the Cauchy-Schwarz inequality and (1.11), to have

3.42 $$\begin{aligned} \begin{aligned} \big |\big ({\widehat{\textsf{A}}}_1,\chi \big )\big | \le&\,C\,\tfrac{h^2}{\tau }\,\Vert u^1-u^0\Vert _2 \,\Vert \chi \Vert \\ \le&\,C\,\tfrac{h^2}{\tau }\,\left( \int _{0}^{t_1} \Vert u_t(s,\cdot )\Vert _2\,ds\right) \,\Vert \chi \Vert ,\\ \end{aligned} \end{aligned}$$

3.43 $$\begin{aligned} \begin{aligned} \big |\big ({\widehat{\textsf{A}}}_2,\chi \big )\big | \le&\,C\,\Vert \eta ^1\Vert \,\Vert \chi \Vert \\ \le&\,C\,h^2\,\Vert u^1\Vert _2\,\Vert \chi \Vert \\ \le&\,C\,h^2\,\Vert \chi \Vert ,\\ \end{aligned} \end{aligned}$$

3.44 $$\begin{aligned} \big |\big ({\widehat{\textsf{A}}}_3,\chi \big )\big | \le \,C\,\Vert \theta _h^1\Vert \,\Vert \chi \Vert \end{aligned}$$

and

3.45 $$\begin{aligned} \begin{aligned} \big |\big ({\widehat{\textsf{A}}}_4,\chi \big )\big | \le&\,\Vert \xi _1\Vert \,\Vert \chi \Vert \\ \end{aligned} \end{aligned}$$

for $\chi \in {\textsf{S}_h}$. Also, we use the Cauchy-Schwarz inequality, (1.11), (3.6), (3.9), (3.5) and (2.7), to obtain

3.46 $$\begin{aligned} \begin{aligned} \big |\big ({\widehat{\textsf{B}}}_1^m,\chi \big )\big | \le&\,C\,\tfrac{h^2}{\tau }\,\big \Vert u^{m}-u^{m-1}\big \Vert _2 \,\Vert \chi \Vert \\ \le&\,C\,\tfrac{h^2}{\tau }\,\left( \int _{t_{m-1}}^{t_m} \Vert u_t(s,\cdot )\Vert _2\,ds\right) \,\Vert \chi \Vert ,\\ \end{aligned} \end{aligned}$$

3.47 $$\begin{aligned} \begin{aligned} \big |\big ({\widehat{\textsf{B}}}_2^m+{\widehat{\textsf{B}}}_{5,4}^m,\chi \big )\big | \le&\,C\,\left( \,\Vert \eta ^m\Vert +\Vert \eta ^{m-1}\Vert \,\right) \,\Vert \chi \Vert \\ \le&\,C\,h^2\,(\Vert u^m\Vert _2+\Vert u^{m-1}\Vert _2) \,\Vert \chi \Vert \\ \le&\,C\,h^2\,\Vert \chi \Vert ,\\ \end{aligned} \end{aligned}$$

3.48 $$\begin{aligned} \big |\big ({\widehat{\textsf{B}}}_3^m+{\widehat{\textsf{B}}}_{5,5}^m,\chi \big )\big | \le \,C\,\left( \Vert \theta _h^{m}\Vert +\Vert \theta _h^{m-1}\Vert \,\right) \,\Vert \chi \Vert , \end{aligned}$$

3.49 $$\begin{aligned} \begin{aligned} \big |\big ({\widehat{\textsf{B}}}_{5,2}^m +{\widehat{\textsf{B}}}_{5,3}^m,\chi \big )\big | \le&\,C\,\Vert u^{m-1}-\textsf{W}_{{\lambda _{\star }},h}^{m-1}\Vert \,\Vert \chi \Vert \\ \le&\,C\,\left( \,\Vert \eta ^{m-1}\Vert +\Vert \theta _h^{m-1}\Vert \,\right) \,\Vert \chi \Vert \\ \le&\,C\,\left( \,h^2\,\Vert u^{m-1}\Vert _2+\Vert \theta _h^{m-1}\Vert \,\right) \,\Vert \chi \Vert \\ \le&\,C\,\left( \,h^2+\Vert \theta _h^{m-1}\Vert \,\right) \,\Vert \chi \Vert \\ \end{aligned} \end{aligned}$$

and

3.50 $$\begin{aligned} \begin{aligned} \big |({\widehat{\textsf{B}}}_4^m+{\widehat{\textsf{B}}}_{5,1}^m,\chi )\big | \le&\,C\,\left( \Vert u^{m}-u^{m-1}\Vert +\Vert \xi _m\Vert \right) \,\Vert \chi \Vert \\ \le&\,C\,\left( \int _{t_{m-1}}^{t_m}\Vert u_t(s,\cdot )\Vert \,ds +\Vert \xi _m\Vert \right) \,\Vert \chi \Vert \\ \le&\,C\,\textsf{Q}_{\scriptscriptstyle {\texttt {LCN}}}^m(u)\,\Vert \chi \Vert \\ \end{aligned} \end{aligned}$$

for $$\chi \in {\textsf{S}_h}$$ and $$m=2,\dots ,N$$.

Part III: Here, we move towards the estimation of $(\theta _h^n)_{n=1}^{\scriptscriptstyle N}$ in the $L^2({\varOmega })$ norm. First, we set $\chi =\theta _h^1$ in (3.39), and then take real parts, to have

3.51 $$\begin{aligned} \Vert \theta _h^1\Vert ^2=\,\tau \,\sum _{\ell =1}^4 \textsf{Re}\big [\big ({\widehat{\textsf{A}}}_{\ell }, \theta _h^1\big )\big ]. \end{aligned}$$

Using (3.51), (3.42), (3.43), (3.45) and the fact that $\textsf{Re}\big [\big ({\widehat{\textsf{A}}}_3,\theta _h^1\big )\big ]=0$, we obtain

$$\begin{aligned} \Vert \theta _h^1\Vert ^2 \le C\,\left( \tau \,\Vert \xi _1\Vert +\tau \,h^2+h^2\,\int _{0}^{t_1} \Vert u_t(s,\cdot )\Vert _2\,ds \right) \,\Vert \theta _h^1\Vert , \end{aligned}$$

which, along (2.7), yields

3.52 $$\begin{aligned} \Vert \theta _h^1\Vert \le \,C\,\left( \,\tau \,\textsf{Q}_{\scriptscriptstyle {\texttt {LCN}}}^1(u) +\tau \,h^2+h^2\,\int _{0}^{t_1} \Vert u_t(s,\cdot )\Vert _2\,ds\,\right) . \end{aligned}$$

Let $m\in \{2,\dots ,N\}$. Setting $\chi ={\mathcal {A}}_{\tau }\theta _h^{m}$ in (3.40), using (3.41) and then taking real parts, we get

3.53 $$\begin{aligned} \Vert \theta _h^m\Vert ^2-\Vert \theta _h^{m-1}\Vert ^2= 2\,\tau \,\sum _{\ell =1}^4\textsf{Re}\left[ \big ({\widehat{\textsf{B}}}_{\ell }^m, {\mathcal {A}}_{\tau }\theta _h^m\big )\right] +2\,\tau \,\sum _{\ell =1}^5 \textsf{Re}\left[ \big ({\widehat{\textsf{B}}}_{5,\ell }^m, {\mathcal {A}}_{\tau }\theta _h^m\big )\right] . \end{aligned}$$

Since $\textsf{Re}\big [\big ({\widehat{\textsf{B}}}_3^m+{\widehat{\textsf{B}}}_{5,5}^m, {\mathcal {A}}_{\tau }\theta _h^m\big )\big ]=0$, after combining (3.53), (3.46), (3.47), (3.49) and (3.50), we arrive at

3.54 $$\begin{aligned} \Vert \theta _h^m\Vert -\Vert \theta _h^{m-1}\Vert \le \,C\,\left( \, \tau \,\Vert \theta _h^{m-1}\Vert +\tau \,\textsf{Q}_{\scriptscriptstyle {\texttt {LCN}}}^m(u)+\tau \,h^2 +h^2\,\int _{t_{m-1}}^{t_m}\Vert u_t(s,\cdot )\Vert _2\,ds\right) . \end{aligned}$$

As a result of (3.34) it holds that $\theta _h^0=0$, and thus (3.54) and (3.52), easily, yield

3.55 $$\begin{aligned} \begin{aligned} \Vert \theta _h^{m}\Vert \le&\,(1+C\,\tau )\,\Vert \theta _h^{m-1}\Vert +C\,Q_{\scriptscriptstyle {\texttt {LCN}}}^m\\ \le&\,e^{C\,\tau }\,\Vert \theta _h^{m-1}\Vert +C\,Q_{\scriptscriptstyle {\texttt {LCN}}}^m,\quad m=1,\dots ,N,\\ \end{aligned} \end{aligned}$$

where

3.56 $$\begin{aligned} Q_{\scriptscriptstyle {\texttt {LCN}}}^m:=\,\tau \,\textsf{Q}_{\scriptscriptstyle {\texttt {LCN}}}^m(u) +\tau \,h^2+h^2\,\int _{t_{m-1}}^{t_m}\Vert u_t(s,\cdot )\Vert _2\,ds. \end{aligned}$$

Finally, applying a standard induction argument on (3.55) (see (1.16)-(1.17)), it follows that

3.57 $$\begin{aligned} \begin{aligned} \max _{1\le {m}\le {\scriptscriptstyle N}}\Vert \theta _h^{m}\Vert \le&\,C\,\sum _{\ell =1}^{\scriptscriptstyle N}Q_{\scriptscriptstyle {\texttt {LCN}}}^{\ell }\\ \le&\,C\,(\tau +h^2).\\ \end{aligned} \end{aligned}$$

Part IV: Now, we focus on the estimation of $(\theta _h^n)_{n=1}^{\scriptscriptstyle N}$ in the $H^1({\varOmega })$ norm. We begin by setting $\chi =\theta _h^1$ in (3.39) and then by taking imaginary parts, to have

$$\begin{aligned} \Vert \nabla \theta _h^1\Vert ^2=\,2\,\sum _{\ell =1}^4 \textsf{Im}\big [\big ({\widehat{\textsf{A}}}_{\ell },\theta _h^1\big )\big ] \le \,2\,\sum _{\ell =1}^4 \big |\big ({\widehat{\textsf{A}}}_{\ell },\theta _h^1\big )\big |, \end{aligned}$$

which, along with (3.42), (3.43), (3.44), (3.45), (2.7) and (3.56), yields

3.58 $$\begin{aligned} \begin{aligned} \Vert \nabla \theta _h^1\Vert ^2\le&\,C\,\Vert \theta _h^1\Vert \,\left[ \Vert \theta _h^1\Vert +\Vert \xi _1\Vert +h^2+\tfrac{h^2}{\tau }\,\int _0^{t_1} \Vert u_t(s,\cdot )\Vert _2\;ds\right] \\ \le&\,C\,\Vert \theta _h^1\Vert \,\left[ \Vert \theta _h^1\Vert +\textsf{Q}_{\scriptscriptstyle {\texttt {LCN}}}^1(u)+h^2+\tfrac{h^2}{\tau }\,\int _0^{t_1} \Vert u_t(s,\cdot )\Vert _2\;ds\right] \\ \le&\,C\,\Vert \theta _h^1\Vert \,\left( \Vert \theta _h^1\Vert +\tfrac{1}{\tau }\,Q_{\scriptscriptstyle {\texttt {LCN}}}^1\right) .\\ \end{aligned} \end{aligned}$$

Let $m\in \{2,\dots ,N\}$. Setting $\chi =\theta _h^{m}-\theta _h^{m-1}$ in (3.40), and then equating imaginary parts, we obtain

$$\begin{aligned} \Vert \nabla \theta _h^m\Vert ^2-\Vert \nabla \theta _h^{m-1}\Vert ^2 =2\,\sum _{\ell =1}^4\textsf{Im}\left[ \left( {\widehat{\textsf{B}}}_{\ell }^m, \theta _h^m-\theta _h^{m-1}\right) \right] +2\,\sum _{\ell =1}^5\textsf{Im}\left[ \left( {\widehat{\textsf{B}}}_{5,\ell }^m, \theta _h^m-\theta _h^{m-1}\right) \right] , \end{aligned}$$

which, along with (3.46), (3.47), (3.48), (3.49), (3.50) and (3.56), yields

3.59 $$\begin{aligned} \begin{aligned} \Vert \nabla \theta _h^m\Vert ^2-\Vert \nabla \theta _h^{m-1}\Vert ^2 \le&\,C\,\Vert \theta _h^{m}-\theta _h^{m-1}\Vert \, \left( \Vert \theta _h^{m}\Vert +\Vert \theta _h^{m-1}\Vert +\tfrac{1}{\tau }\,Q_{\scriptscriptstyle {\texttt {LCN}}}^m\right) \\ \le&\,C\,\left( \Vert \theta _h^{m}\Vert +\Vert \theta _h^{m-1}\Vert \right) \, \left( \Vert \theta _h^{m}\Vert +\Vert \theta _h^{m-1}\Vert +\tfrac{1}{\tau }\,Q_{\scriptscriptstyle {\texttt {LCN}}}^m\right) .\\ \end{aligned} \end{aligned}$$

Since $\theta _h^0=0$, in light of (3.59), (3.58) and (3.57), we arrive at

$$\begin{aligned} \Vert \nabla \theta _h^m\Vert ^2-\Vert \nabla \theta _h^{m-1}\Vert ^2 \le \,C\,(\tau +h^2)\,\left( \tau +h^2+\tfrac{1}{\tau }\,Q_{\scriptscriptstyle {\texttt {LCN}}}^m\right) , \quad m=1,\dots ,N, \end{aligned}$$

which, after summing with respect to *m*, yields

3.60 $$\begin{aligned} \begin{aligned} \max _{1\le {m}\le {\scriptscriptstyle N}}\Vert \nabla \theta _h^m\Vert \le&\,C\,\left[ \,\tau ^{-1}(\tau +h^2)^2 +\left( 1+\tfrac{h^2}{\tau }\right) \,\sum _{\ell =1}^{\scriptscriptstyle N}Q_{\scriptscriptstyle {\texttt {LCN}}}^{\ell }\right] ^{\frac{1}{2}}\\ \le&\,C\,\left[ \,\tau ^{-1}(\tau +h^2)^2 +\left( 1+\tfrac{h^2}{\tau }\right) \,(\tau +h^2)\right] ^{\frac{1}{2}}\\ \le&\,C\,\left[ \,\left( 1+\tfrac{h^2}{\tau }\right) \,(\tau +h^2)\right] ^{\frac{1}{2}}\\ \le&\,C\,\left( \tau +h^2+\tfrac{h^4}{\tau }\right) ^{\frac{1}{2}}\\ \le&\,C\,\left[ \tau ^{\frac{1}{2}}+h\,\left( 1+\tfrac{h^2}{\tau } \right) ^{\frac{1}{2}}\right] .\\ \end{aligned} \end{aligned}$$

In addition to the estimate above, after applying (1.12) on (3.57), we obtain

3.61 $$\begin{aligned} \max _{1\le {m}\le {\scriptscriptstyle N}}\Vert \nabla \theta _h^{m}\Vert \le \, C\,\left( h+\tfrac{\tau }{h}\right) . \end{aligned}$$

Combining (3.61) and (3.60) (cf. [20]), we conclude that

3.62 $$\begin{aligned} \max _{1\le {m}\le {\scriptscriptstyle N}}\Vert \nabla \theta _h^{m}\Vert \le \,C\,\left\{ \begin{aligned}&\,2\,h,\hspace{41.25641pt}\text {if}\quad \sqrt{\tau }\le h\\&\,\tau ^{\frac{1}{2}}+h\,\sqrt{2}, \quad \text {if}\quad h\le \sqrt{\tau }\\ \end{aligned}\right. \quad \le \,C\,(\tau ^{\frac{1}{2}}+h). \end{aligned}$$

Part V: Combining (1.13), (1.15), (3.62) and (3.57) (cf. (3.33)), we conclude that there exists a positive constant $\textsf{C}_{\texttt {LCN}}$ such that

$$\begin{aligned} \begin{aligned} |\textsf{W}_{{\lambda _{\star }},h}^{m}|_{\infty } \le&\,\textsf{C}_{\texttt {LCN}}\,\left[ \varphi _{\star }(d,h)\, (\tau ^{\frac{1}{2}}+h)+h^{2-\frac{d}{2}}\right] +\tfrac{{\lambda _{\star }}}{6}, \quad m=1,\dots ,N. \end{aligned} \end{aligned}$$

Thus, assuming that $\textsf{C}_{\texttt {LCN}}\,\left[ \varphi _{\star }(d,h)\, (\tau ^{\frac{1}{2}}+h)+h^{2-\frac{d}{2}}\right] \le \tfrac{{\lambda _{\star }}}{6}$, it follows that

$$\begin{aligned} \max \limits _{1\le {m}\le {\scriptscriptstyle N}} |\textsf{W}_{{\lambda _{\star }},h}^m|_{\infty } \le \,\tfrac{2\,{\lambda _{\star }}}{6}<{\lambda _{\star }}, \end{aligned}$$

which, in light of (3.3), yields $\textsf{G}_{{\lambda _{\star }}}(\textsf{W}_{{\lambda _{\star }},h}^m)=\textsf{W}_{{\lambda _{\star }},h}^m$ for $m=1,\dots ,N$. The latter equality, along with (1.22)-(1.24) and (3.34)-(3.36), yields $\textsf{W}_{{\lambda _{\star }},h}^m=\textsf{W}_h^m$ for $m=0,\dots ,N$, and consequently (3.38) follows easily from (3.57), (3.62) and (1.11).

## Numerical Results

We implemented the (LBEFE) and (LCNFE) methods in a Python 3.7.0 program, where the resulting linear systems of algebraic equations are solved by applying the subroutine gmres of the library scipy.sparse.linalg, which is an implementation of the usual Generalized Minimum Residuals (GMRES) method. Also, to compute the elliptic projection of the initial condition $u_0$, we solve the corresponding linear system of algebraic equations by applying the subroutine cg of the latter library, which is based on the Conjugate Gradient (CG) method.

When the exact solution to the problem is given, we compute the numerical approximations errors

$$\begin{aligned} \textsf{E}_{\texttt {DL2}}(N,h):=\max \limits _{1\le {n}\le {\scriptscriptstyle N}} \,\Vert \textsf{U}_h^n-u^n\Vert \quad \text {and}\quad \textsf{E}_{\texttt {DH1}}(N,h):=\max \limits _{1\le {n}\le {\scriptscriptstyle N}} \,\Vert \textsf{U}_h^n-u^n\Vert _{\scriptscriptstyle 1}, \end{aligned}$$

where the norm-integrals are approximated by employing a numerical quadrature technique based on a high order Gauss quadrature rule. Choosing $h=\textsf{h}(N)$, where $\textsf{h}(N):=q\,\,N^{-\nu }$ with $q,\nu >0$, we compute the experimental order of convergence $\textsf{R}(N_1,N_2)$ with respect to $N^{-1}$, corresponding to given values $N_1$ and $N_2$ of *N*, by

$$\begin{aligned} \textsf{R}(N_1,N_2):=\tfrac{ \ln \left[ \textsf{E}\left( N_1,\textsf{h}(N_1)\right) / \textsf{E}\left( N_2,\textsf{h}(N_2)\right) \right] }{\ln (N_2/N_1)}, \end{aligned}$$

where $$\textsf{E}=\textsf{E}_{\texttt {DL2}}$$ or $$\textsf{E}_{\texttt {DH1}}$$.

### Example 1

Let $d=1$, $T=1$, ${\varOmega }=(-1,1)$, $V\equiv 0$, $\beta =\frac{1}{4}$ and $\mu =1$. The initial condition $u_0$ and the load *f* are such that the function

$$\begin{aligned} u(t,x)=\tfrac{1}{4}\,\big (t-\tfrac{1}{2}\big )^2\, \textsf{sgn}\big (t-\tfrac{1}{2}\big )\,(1-x^2)\,x^2\,\textsf{sgn}(x) \end{aligned}$$

to be the exact solution to the problem (1.1)-(1.5). Tables 1 and 2 contain the numerical approximation error in the $L^2({\varOmega })$ and the $H^1({\varOmega })$ norms, along with the corresponding experimental orders of convergence for the (LBEFE) and (LCNFE) methods, respectively. In particular, since $\tau =\frac{1}{N}$, by choosing $h=2\,\tau ^{\frac{1}{2}}$ (i.e. $\textsf{h}(N)=2\,N^{-\frac{1}{2}}$) we conclude that the experimental order of convergence in the $L^2({\varOmega })$ norm is equal to 1 with respect to $\tfrac{1}{N}$. However, by setting $h=2\,\tau$ (i.e. $\textsf{h}(N)=\tfrac{2}{N}$), we observe an experimental order of convergence equal to 1 in the $H^1({\varOmega })$ norm for both methods, which is higher than that we obtain by our error analysis.

**Table 1:** Example 1. Experimental order of convergence for the (LBEFE) method

*N*	$\textsf{E}_{\texttt {DL2}}(N,2\,N^{-\frac{1}{2}})$	Rate	$\textsf{E}_{\texttt {DH1}}(N,2\,N^{-1})$	Rate
20	5.925(-3)	—	8.571(-3)	—
40	3.229(-3)	0.87	4.768(-3)	0.84
80	1.977(-3)	0.70	2.510(-3)	0.92
160	9.407(-4)	1.07	1.288(-3)	0.96
320	4.825(-4)	0.96	6.529(-4)	0.98
640	2.334(-4)	1.04	3.286(-4)	0.99

**Table 2:** Example 1. Experimental order of convergence for the (LCNFE) method

*N*	$\textsf{E}_{\texttt {DL2}}(N,2\,N^{-\frac{1}{2}})$	Rate	$\textsf{E}_{\texttt {DH1}}(N,2\,N^{-1})$	Rate
20	5.809(-2)	—	8.335(-2)	—
40	2.809(-2)	1.04	4.179(-2)	0.99
80	1.625(-2)	0.78	2.091(-2)	0.99
160	7.367(-3)	1.14	1.045(-2)	0.99
320	3.694(-3)	0.99	5.229(-3)	0.99
640	1.717(-3)	1.10	2.614(-3)	0.99

### Example 2

Let $d=2$, $T=1$, ${\varOmega }=(-1,1)\times (-1,1)$, $\mu =1$, $\beta =\tfrac{1}{2}$ and the rough potential (see Section 6 in [21])

$$\begin{aligned} V(x):= \left\lfloor 5 + 2\sin (\tfrac{\pi x_1}{3}) \sin (\tfrac{\pi x_2}{3}) \right\rfloor \quad \forall \,x\in {\overline{{\varOmega }}}, \end{aligned}$$

where $\lfloor \cdot \rfloor$ denotes the floor function. As usual, we determine the initial condition $u_0$ and the load *f* by considering that the function

$$\begin{aligned} u(t,x) = \tfrac{1}{2}\exp \left( \tfrac{\textrm{i}}{2} \left( t - \tfrac{1}{2}\right) ^2 \textsf{sgn}\left( t - \tfrac{1}{2}\right) \right) \,(1 - x_1^2)\,(1 - x_2^2)\, \left[ x_1^2\,\textsf{sgn}(x_1)+x_2^2\,\textsf{sgn}(x_2)\right] \end{aligned}$$

is the exact solution to (1.2)-(1.4). We post on Tables 3 and 4 the corresponding computational errors and experimental rates of convergence in the $L^2({\varOmega })$ and the $H^1({\varOmega })$ norms for the (LBEFE) and (LCNFE) methods, respectively. In particular, by choosing $h=\tau ^{\frac{1}{2}}$ we conclude a first order convergence in the $L^2({\varOmega })$ norm with respect to $\tau$ which is in agreement with our error estimates. Also, setting $h=\tau$ we get again a first order convergence in the $H^1({\varOmega })$ norm with respect to $\tau$ which is higher than that have shown in our analysis. Moreover, we observe that both methods are able to handle the case of a rough potential where $V\in L^{\infty }({\varOmega })$.

**Table 3:** Example 2. Experimental order of convergence for the (LBEFE) method

*N*	$\textsf{E}_{\texttt {DL2}}(N,\,N^{-\frac{1}{2}})$	Rate	$\textsf{E}_{\texttt {DH1}}(N,\,N^{-1})$	Rate
10	4.455(-2)	—	1.128(-1)	—
20	2.293(-2)	0.95	5.612(-2)	1.00
40	1.241(-2)	0.88	2.814(-2)	0.99
80	6.102(-3)	1.02	1.411(-2)	0.99
160	3.198(-3)	0.93	7.076(-3)	0.99
320	1.693(-3)	0.91	3.543(-3)	0.99

**Table 4:** Example 2. Experimental order of convergence for the (LCNFE) method

*N*	$\textsf{E}_{\texttt {DL2}}(N,\,N^{-\frac{1}{2}})$	Rate	$\textsf{E}_{\texttt {DH1}}(N,\,N^{-1})$	Rate
10	4.589(-2)	—	1.129(-1)	—
20	2.162(-2)	1.08	5.604(-2)	1.01
40	1.116(-2)	0.95	2.802(-2)	0.99
80	5.245(-3)	1.08	1.404(-2)	0.99
160	2.622(-3)	0.99	7.034(-3)	0.99
320	1.310(-3)	1.00	3.520(-3)	0.99

### Example 3

**Fig. 1:**
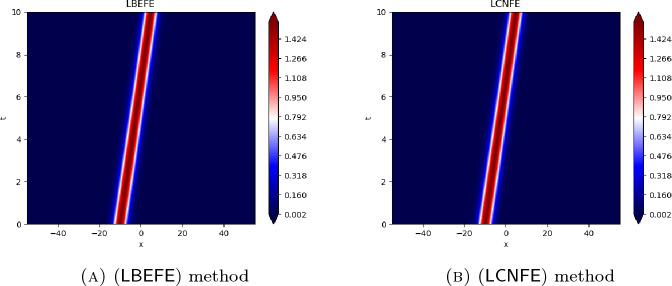
Space-time contour plots of the approximation of |*u*| in Example 3

Let $\mu =1$, $V\equiv 0$ and $f=0$. Then a soliton solution to (1.2) is given by

$$\begin{aligned} u_{\texttt {S}}(t,x):=\left( \tfrac{2}{\beta } \left( 1 + \tfrac{2}{\beta }\right) \right) ^{\frac{1}{\beta }} \,\eta ^{\frac{2}{\beta }}\,e^{\textrm{i}\,(\kappa \,x+(\frac{4\,\eta ^2}{\beta ^2}-\kappa ^2)\,t)} \,\textrm{sech}^{\frac{2}{\beta }} \left( \eta \,(x-2\,\kappa \,t-\rho )\right) \quad \forall \,(t,x)\in [0,+\infty )\times {\mathbb {R}}, \end{aligned}$$

where $\eta$, $\rho$, $\kappa$ and $\beta \in (0,1)$ are real numbers, and which generalizes that formulated in [30] for $\beta =\tfrac{1}{2}$ (cf. (3) in [30]). Choosing $T=10$, ${\varOmega }=(-55,55)$, $\beta =\tfrac{1}{2}$, $\eta =0.25$, $\kappa = 0.75$, $\mu =1$ and $\rho = -10$, $u_{\texttt {S}}$ has almost compact support on $[0,T]\times {\varOmega }$ and thus we can consider it as the solution to the problem (1.2)-(1.4) with initial condition $u_0(x)=u_{\texttt {S}}(0,x)$ for $x\in {\overline{{\varOmega }}}$. In the numerical experiments we compute approximations of $u_{\texttt {S}}$, posting the corresponding computational errors on Tables 5 and 6 for the (LBEFE) and (LCNFE) methods, respectively. Moreover, we present in Figure 1 the space-time contour plot of the absolute value of the approximation obtained by using the (LBEFE) and (LCNFE) methods, for $N=10000$ and $h = \tfrac{11}{500}=0.022$.

**Table 5:** Example 3. Experimental order of convergence for the (LBEFE) method

*N*	$\textsf{E}_{\texttt {DL2}}(N,11\,N^{-\frac{1}{2}})$	Rate	$\textsf{E}_{\texttt {DH1}}(N,110\,N^{-1})$	Rate
640	7.894(-1)	—	5.024(-1)	—
1280	4.131(-1)	0.93	2.306(-1)	1.12
2560	2.068(-1)	0.99	1.103(-1)	1.06
5120	1.036(-1)	0.99	5.394(-2)	1.03

**Table 6:** Example 3. Experimental order of convergence for the (LCNFE) method

*N*	$\textsf{E}_{\texttt {DL2}}(N,11\,N^{-\frac{1}{2}})$	Rate	$\textsf{E}_{\texttt {DH1}}(N,110\,N^{-1})$	Rate
640	6.185(-1)	—	1.975(-1)	—
1280	3.232(-1)	0.93	9.743(-2)	1.01
2560	1.601(-1)	1.01	5.110(-2)	0.93
5120	7.985(-2)	1.00	2.645(-2)	0.94

### Example 4

Let $d=1$, $T=5$, ${\varOmega }=(-55,55)$, $\beta =\tfrac{1}{2}$, $\mu =1$, $f=0$ and the initial soliton values (cf. Section III in [30])

$$\begin{aligned} u_0^{[\ell ]}(x) = 400\,\,\eta ^4\,\, e^{\textrm{i}\,\kappa _{\ell }\,x} \,\,\textrm{sech}^4\left( \eta \,(x-\rho _{\ell })\right) \quad \forall x\in \overline{{\varOmega }},\quad \ell = 1,2. \end{aligned}$$

**Fig. 2:**
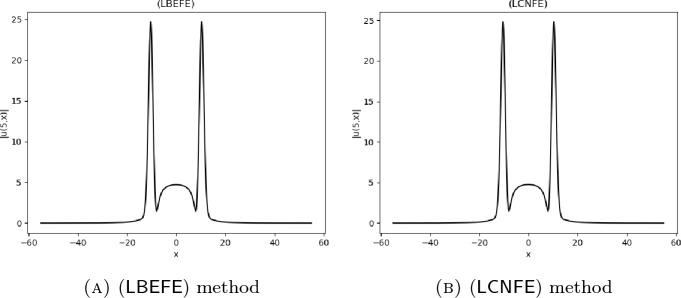
Snapshots of the (LBEFE) and (LCNFE) methods at $t=5$ in Example 4

**Fig. 3:**
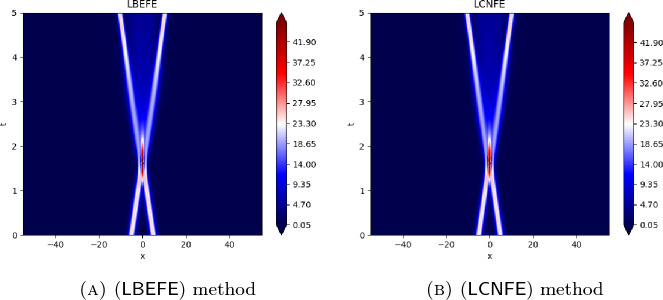
Space-time contour plots of the approximation of |*u*| in Example 4

Then, for $\rho _{1}=5$, $\rho _{2}=-5$, $\eta =\tfrac{1}{2}$ and $\kappa _1=-\kappa _2=\tfrac{3}{2}$, we consider the initial condition

$$\begin{aligned} u_0(x) = u_0^{[1]}(x) + u_0^{[2]}(x)\quad \forall \,x\in \overline{{\varOmega }}, \end{aligned}$$

in order to observe the dynamical behavior of the solution, when the two solitons collide. In Figure 2, we post the snapshots of the numerical solution obtained at the final time, and in Figure 3 the space-time contour plot of the approximation of |*u*|, when we use the (LBEFE) and (LCNFE) methods for $N=20000$ and $h=0.01$.

## Conlusions

We approximate the solution to a nonlinear Schrödinger equation with a Schamel-type nonlinearity, by the (LBEFE) method which is dissipative (see Remark 1.3) and the (LCNFE) method which is conservative (see Remark 1.5). The latterly mentioned methods are linearly implicit and at each time-step the solution of a linear system of algebraic equation is required, the matrix of which depends on the finite element basis chosen. For both methods, an optimal order $O(\tau +h^2)$ error estimate in the $L^2({\varOmega })$ norm is provided. Moreover, an $O(\tau ^{\alpha }+h)$ error estimate in the $H^1({\varOmega })$ norm is established, where $\alpha =\frac{3}{4}$ for the (LBEFE) method, and $\alpha =\frac{1}{2}$ for the (LCNFE) method. The error estimation became possible by estimating the approximation error of a modified version of the proposed methods, and requires no CFL conditions for $d=1$, and a mild mesh condition of the form $\big (h^{\frac{2-d}{2}}\,|\ln (h)|^{\frac{d-1}{d}} \,\tau ^{\alpha }+h^{2-\frac{d}{2}}\big )=O(1)$ for $d\in \{2,3\}$. The results are new, and it is the first time in the literature that the use of the general conforming finite element method for the approximation of the solution to the nonlinear Schrödinger equation with a Schamel-type nonlinearity is addressed. Results from numerical experiments for both methods confirm their expected order of the $L^2({\varOmega })$-convergence and expose their performance. However, we were not able to verify experimentally the suboptimal order of the $H^1({\varOmega })$-convergence shown in the theory. Moreover, from the numerical results shown, we observe that the (LCNFE) method seems to be more efficient than the (LBEFE) method. The convergence analysis framework we developed here, can be also used to provide analogous error estimates for other linearly implicit finite element methods based on a non locally Lipschitz linearization of *g* (see, e.g. [4, 19]). Future research plans focus on the investigation of the convergence of higher order numerical methods with preserving properties.

## Data Availability

Inquiries about data availability shall be directed to the authors.
